# The innovative dimension of the research training programmes under H2020-MSCA-ITN: a methodological approach to track, measure and analyse innovative aspects and provide policy-feedback conclusions.

**DOI:** 10.12688/f1000research.138482.2

**Published:** 2023-10-26

**Authors:** Ioannis Bitsios, Fabrizio Martone, Riccardo Ricci, Audrey Arfi

**Affiliations:** 1 REA.A1 Marie Sklodowska-Curie Actions - Doctoral Networks, European Research Executive Agency, Brussels, 1049, Belgium

**Keywords:** Innovation, Policy-feedback, H2020, Marie Sklodowska-Curie Actions -Innovative Training Networks (MSCA-ITN), Indicators, Methodology

## Abstract

Innovative research training programmes funded by the European Union are essential for the forging of highly skilled researchers to tackle, via breakthrough ideas and solutions, the challenges of our society. Being able to track, measure and analyse innovative aspects of the Marie Sklodowska-Curie Actions, Innovative Training Networks under the Horizon2020 funding scheme enables the impact assessment of such programmes, while filtering best practices and the generated knowledge that could ultimately breed and create further innovation. In parallel, it helps the identification of areas for improvement, the understanding of new needs to be accommodated and the co-design and implementation of EU funding policy activities to further promote innovation and excellence for researchers across Europe and beyond. In this study, a novel methodological approach is proposed for tracking and analysing innovation, using a representative sample of projects. Basic innovation indicators are examined and considered from the existing literature and from the applicable Multi-Annual Framework Programme Horizon2020. Additional ones are defined, complemented by questionnaires/surveys findings, to capture innovative aspects for which the standard indicators do not apply. Data mining and data visualization tools are used for the collection and processing of data. Innovation Radar ^2^ (IR) reports and HorizonResultsBooster ^3^ services are also engaged for the cross-validation of the identified innovative aspects. The study provides first-level input for policy-feedback activities, by identifying scientific domains and EU countries that may potentially require more attention for innovation generation. It highlights domains that are front-runners and can be used as examples or best practices for under-represented domains in terms of innovative outputs. Collaboration with organisations, defined as medium/high innovators, can increase innovation generation and success in future projects. Best practices are collected to serve as references for designing impactful future training programmes. The excellence of the H2020-MSCA-ITN actions is confirmed via the generated innovations.

## Introduction

The H2020 MSCA Innovative Training Networks ^4^ aim to train a new generation of creative, entrepreneurial and innovative Early-Stage Researchers (ESRs), able to face current and future challenges and to convert knowledge and ideas into products and services for the economic and social welfare of society.

H2020-MSCA-ITN has the objective to foster excellence and structure research and doctoral training in Europe (EU Member States and H2020 Associated Countries), extending the traditional academic research training setting, incorporating elements of Open Science ^5^ and equipping researchers with the right combination of research-related and transferable competences. It aims to provide enhanced career prospects in both the academic and non-academic sectors through international, interdisciplinary and intersectoral mobility combined with an innovation-oriented mind-set. The H2020-MSCA-ITN programme encompasses three different implementation modalities: The European Training Networks (ETNs), the European Industrial Doctorates (EIDs), and the European Joint Doctorates (EJDs).

The H2020-MSCA-ITN calls for proposals were highly competitive, as demonstrated by the high number of applications submitted every year (more than 1500 submitted proposals on average) and a low success rate, ranging from 7-8% to 12% depending on the year and the scientific domain. From 2014 to 2020, 1035 H2020-MSCA-ITN project grants were signed, out of which 78% were ETNs, 15% were EIDs and 7% were EJDs. Funded H2020-MSCA-ITN projects represented a total budget of 3,42 billion EUR, i.e. more than 55% of the total Marie Sklodowska-Curie Actions (MSCA) programme budget and almost 4,4% of the total H2020 framework programme budget. So far, more than 10600 doctoral candidates were recruited and trained within the H2020-MSCA-ITN projects.

The set of these networks potentially represents a gold mine of generated innovation, success stories, best and innovative practices.

The main goals of this research study were: i) to propose a practical methodology for tracking and measuring innovation in H2020-MSCA-ITN projects, which can also be replicated in other similar Research and Innovation (R&I) portfolios; ii) to collect and present in a comprehensive manner the most relevant innovative elements of H2020-MSCA-ITN projects; iii) to provide insights on projects’ performance with regards to innovation in order to draw relevant policy-related conclusions and recommendations.

## Background

The European Union (EU) is constantly supporting the fostering and generation of innovation in order to respond to the challenges of our era. As presented in the recently published, ‘innovation score board’ ( European Innovation Scoreboard, 2022), the EU is improving its innovation performance year by year. For example, in 2022, the EU overtook Japan and closed the gap with some other global competitors (including Australia, Canada, South Korea and the United States). Thus, measuring innovation is key for the creation and implementation of research and innovation-friendly policies that can further boost innovation creation within the EU. H2020-MSCA-ITN, being the EU’s flagship scheme for establishing transnational doctoral programmes, is of interest to observe their contribution to the long-term innovation ambitions of the Union. The essential role of the innovation performance measuring is clearly expressed in the ‘Science, Research and Innovation Performance of the EU’ (SRIP) report, as a means for further improvement and design of new R&I policies for the EU’s economic growth and sustainable development ( SRIP, 2022).

Without a suitable methodology to track and analyse the potentially generated innovation, a proper project and portfolio analysis is restricted. And most importantly, the measuring of generated innovation becomes difficult which impedes efforts to track the evolution of innovation performance and to provide policy-feedback conclusions. Therefore, being able to monitor innovative performance in EU-funded projects (in this case H2020-MSCA-ITN) is very relevant for research-promotion agencies in order to set ( Taques, López, Basso, Areal (2020)) and - where appropriate - regularly update funding-targeted criteria.

Beyond the advancement of the current state of the art and the excellence in science in several disciplines (bottom-up approach), the H2020-MSCA-ITN action promotes synergies, collaboration, joint supervision and single or joint doctoral programmes, inter-sectoral and international secondments for enhancing the professional experience and skills of recruited Early-Stage Researchers (ESRs), as well as trainings and joint dissemination and outreach activities. Therefore, when measuring innovative performance in H2020-MSCA-ITN, not only ‘technological’ innovation should be considered. ‘Technological’ innovation is considered tangible (essentially can be quantified and measured) and it is potentially generated as a result of the individual research projects that are undertaken by the recruited researchers (ESRs) as well as from the project consortium as a whole. Other non-tangible innovative aspects (qualitative elements being more difficult to quantify or measure) which are inherent to the very nature of the H2020-MSCA-ITN action, are also considered. Such non-tangible innovative elements can be social innovation such as the creation of novel solutions to the needs of the recruited researchers and/or project community, other signature features of the MSCA programme, such as mobility that creates diffusion and transfer of knowledge ( Science, Research and Innovation Performance of the EU, 2022), the cross-sectoral cooperation ( European research & innovation days, 2021), the quality of the supervision and doctoral training, the implementation of secondments in the most efficient and rewarding way for the highly skilled researchers ( Report of Mapping Exercise on Doctoral Training in Europe, 2011) and interesting best practices that H2020-MSCA-ITN projects and participants have put in place to promote innovation in doctoral education ( LERU advice paper, 2014).

One of the starting points to track and measure innovation is to define and list the appropriate indicators. In the literature, there are several references to such indicators which may vary depending on the applied domain (e.g. scientific, technological, industrial, commercial etc.). A notable publication is the ‘Oslo Manual’ ( OECD, European Union (2018)), which comprises guidelines for collecting, reporting and using data on innovation. Another source of inspiration are the H2020 indicators used to assess the results and impact of the programme ( European Commission, Directorate-General for Research and Innovation, 2015). Such indicators include, for example, the number of peer-reviewed publications in high-impact journals, patent applications or awarded ones and less tangible indicators like the cross-country circulation of researchers (for H2020-MSCA-ITN the so-called ‘secondments’).

Regarding tangible indicators, for the currently established metrics for publications, the latest developments on the approach of the European Commission (EC) for research assessment ( European Commission, Directorate-General for Research and Innovation, Coalition for Advancing Research Assessment, 2022), alleviate the mere focus on metrics like high-impact journals and instead promote quality and openness to better assess the impact and value of generated publications. As such, in the methodology that is proposed for our analysis, we went beyond a narrow set of quantitative journal- and publication-based metrics. This is in line with the ‘San Francisco Declaration on Research Assessment’ ( DORA, 2012), the ‘The Leiden Manifesto for research metrics’ ( Bibliometrics: The Leiden Manifesto for research metrics, 2015) and more recently with the Open Science mandate ( The EU’s open science policy, 2021). We also considered non-structured or qualitative sources (i.e. information coming from periodic reports, deliverables and other non-tangible innovation elements), in order to capture to the maximum feasible extent, the generated innovation by the H2020-MSCA-ITN projects.

Regarding less tangible indicators like secondments (or mobility of ESRs), a good source of inspiration for the execution of this analysis was a recent survey launched over the summer of 2020 to nearly 17.000 Marie Curie alumni association (MCAA) members, with over 2.000 respondents completing the survey ( Marie Curie alumni association survey 2020: results). Based on this survey, following an inquiry about career mobility and, in particular how many times researchers moved due to their career, it was possible to verify that only 3.4% of the respondents didn’t change their country of residence while 96.6% respondents reported to have moved at least once due to their careers. The majority moved (51,4% or 947 respondents) between two and three times. Since mobility is an integral part of H2020-MSCA-ITN projects and based on this survey findings, the secondments (or mobility of ESRs) were also considered in this analysis as part of the generation and diffusion cycle of innovative aspects in the H2020-MSCA-ITN projects.

Accordingly, there are already several defined indicators, articles, studies, and reports elaborating on innovation. For this study, all the aforementioned sources were considered in order to tailor and use the suitable set of indicators (or metrics) and propose the methodology for identifying, measuring and understanding innovation generated by the H2020-MSCA-ITN EU-funded projects. With the proposed methodology, the aim is also to highlight innovative aspects of H20202-MSCA-ITN actions beyond particular success stories or significant project results, but rather in a more systematic approach that can bring more findings to the surface. Subsequently and based on these findings, the methodology allows to draw important conclusions which can directly support feedback to policy and the institutional decision-making process in general.

## Methods

### Methods outline

We identified the appropriate population (sample) of H2020-MSCA-ITN projects to be analysed for innovation outputs, and then the nature and type of data to be collected for the analysis as well as the best way to collect the necessary data. Based on the literature analysis, we proposed and defined suitable indicators (KPIs and metrics) in order to track, analyse and present in a quantifiable and qualitative manner innovative elements in the H2020-MSCA-ITN projects.

A first analysis was done on the full H2020-MSCA-ITN portfolio covering the calls from 2014-2020. The source that we used to make the first extraction of data was the EC CORDA database (SAP BusinessObjects Business Intelligence, universe: EGRANTS context: H2020). Since the purpose of the study was to track innovation and similar outputs that have been already generated by the projects, it was decided to narrow the analysis on the population of projects under the calls 2014-2017. Projects under H2020-MSCA-ITN have a normal duration of 4 years (48 months) and with the selected sample of projects from the calls 2014-2017, the purpose was to analyse generated results from the already closed or in the closing mode projects (the analysis started in October 2021). In the closed projects, the interim and final reporting had already been conducted (contractual requirement), which ensured that the generated innovations and similar results were encoded in the EC corporate systems (databases) in order to allow their retrieval and further analysis.

We proceeded to the analysis of the H2020-MSCA-ITN projects from calls 2014-2017, again using the CORDA database/tool. From the extracted population of projects, a further analysis was carried out in order to identify those projects that appeared to have generated or declared innovation. By ‘generated innovation’ we refer to innovation and patents declared by the projects (and encoded in a structured manner in the CORDA database, following the project closure).

Finally, for the projects from calls 2014-2017, an analysis using the defined metrics for tracking and measuring generated innovation, has been performed on a set of data and documents comprising the Horizon Dashboard ^6^ reports, the available H2020-MSCA-ITN project reports and relevant documents (i.e. progress, periodic and final project reports, Expert or Project Officer Assessment reports, ESRs’ questionnaires ^7^ …) for tracking non-tangible innovation, and a set of EC corporate tools and services like databases (CORDA, CORDIS ^8^), the Qlik Sense ^9^ graphical data representation tool, the IR platform and the HRB service.

To summarize, the analysis of the extracted sample of H2020-MSCA-ITN projects was performed using:

1. The reported patents and innovation outputs from CORDA and Qlik Sense (HorizonDashboard).
2. The Innovation Radar (IR) platform and innovation capacity indicators.
3. The use of HRB Service 4 ^10^ (Module A: publishable reports of R&I project clusters analysis) as a proof of concept of the methodology from external independent experts.
4. The analysis of most frequently found terms (word clouds) which can be retrieved from the generated publications.
5. The non-tangible innovation and best practices results which can be retrieved by the non-structured data from the different project reports and documents.
6. Questionnaires/surveys from the ESRs that participated in those projects.

1. Reported patents and innovation outputs (CORDA and Qlik Sense/HorizonDashboard)

We used the CORDA database and the Qlik Sense platform tool for the data extraction. The extracted data were in the form of Microsoft Excel ^@^ spreadsheets.

H2020-MSCA-ITN projects have been generated via 7 calls (1 call per year) that have been performed under the seven years of the H2020 MFF (calls 2014-2020). The H2020-MSCA-ITN portfolio comprises 1034 executed (on-going or closed) projects. A focus on the 2014-2017 calls was considered as more relevant in order to ensure generated innovation from the closed or in the closing mode projects.

Based on the 2014-2017 projects portfolio, a data extraction was done from CORDA database in order to retrieve those projects that self-declared the generation of innovations, including the filing of patents. The extraction of this information was done based on declared innovation and patents, using similar indicators that were provided by the data fields and filters of the CORDA database interface. In the data extraction, we included information fields related to - among others - the project number, call-id, mode, acronym, status, number and description of innovations, number and description of patents and number of publications (see Figure 1.a.1).

**Figure 1.a.1.**
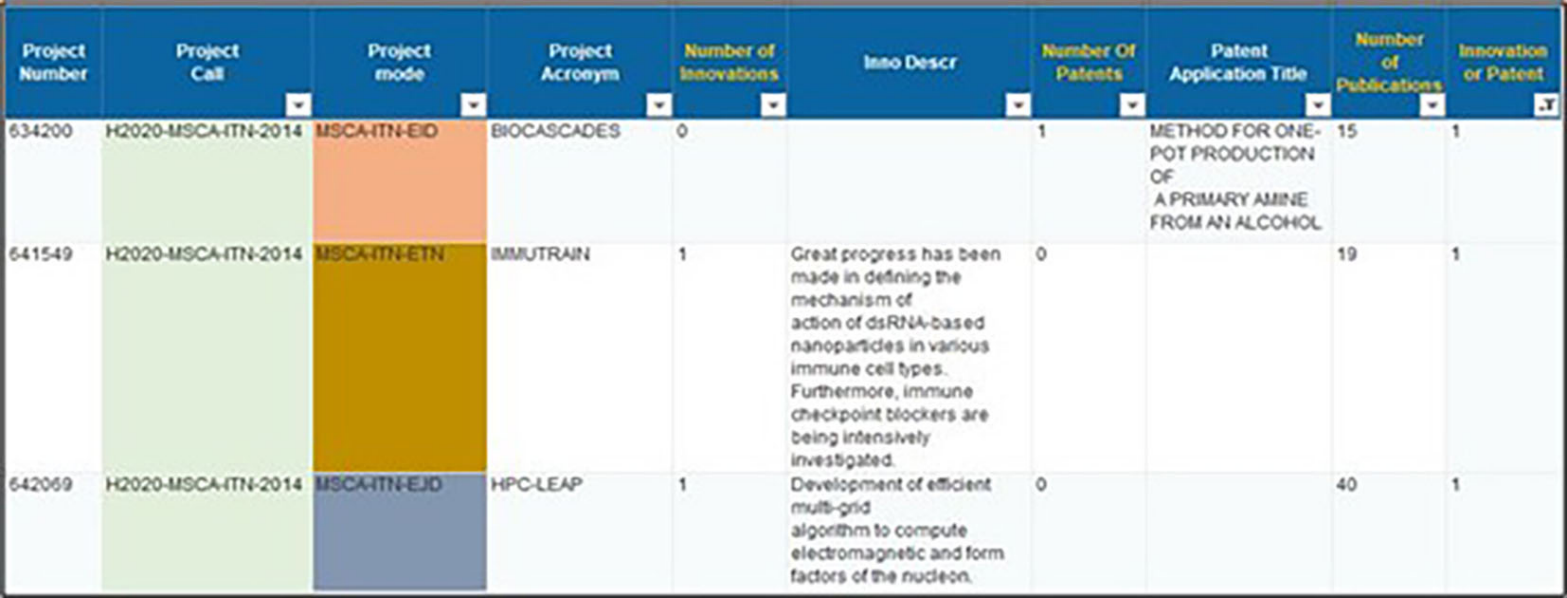
Example of data fields extracted from the CORDA database for 2014-2017 projects.

**Figure 0.1.**
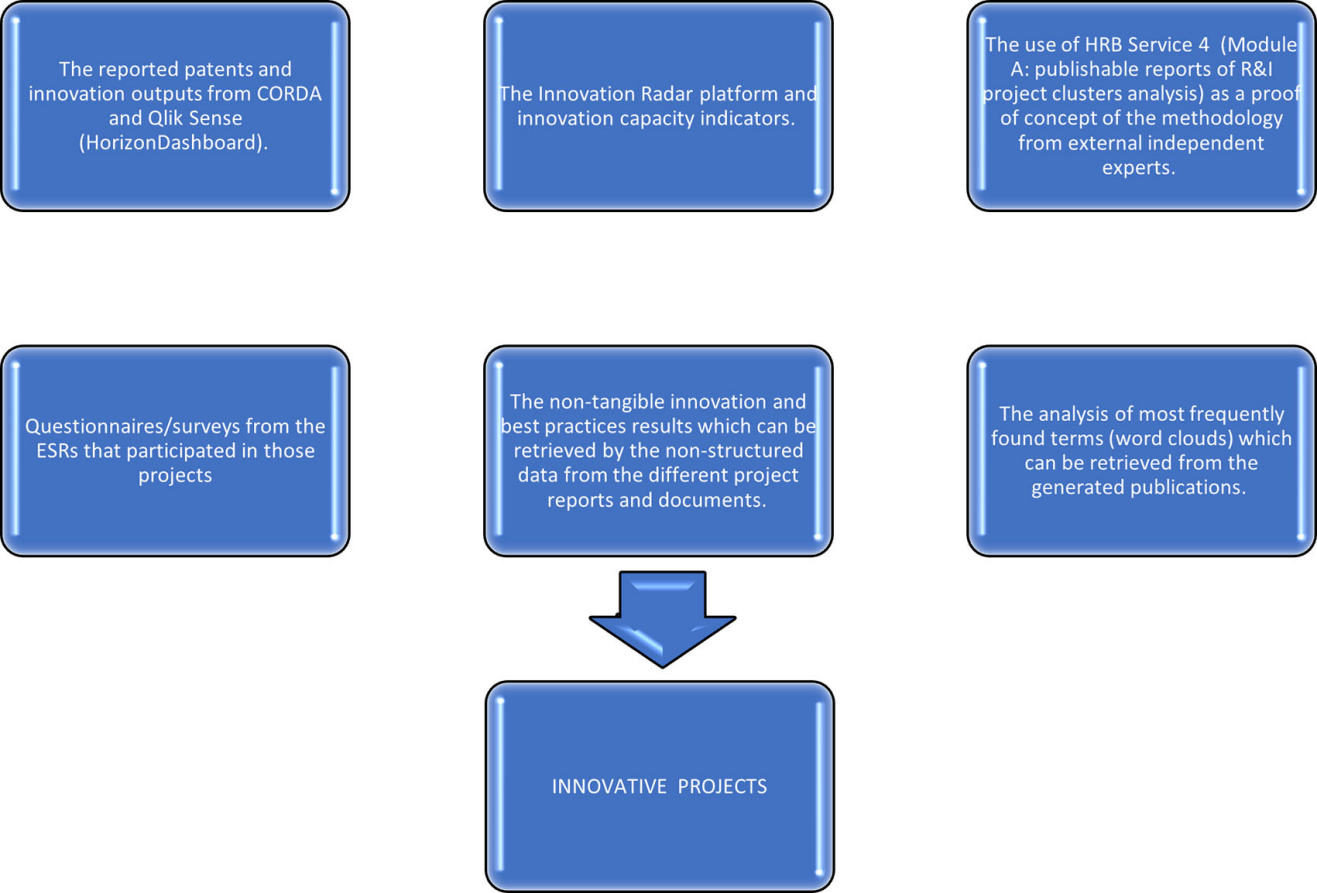
Steps/components of the methodology.

From the extracted data, a filtering of the projects was done based on declared innovation(s) and/or patent(s), resulting in 118 retained projects ( MSCA ITN 2014-2017 innovation and patents [Data set], 2023a).

2. The Innovation Radar (IR) platform and innovation capacity indicators.

In order to further validate that the selection of 118 projects was suitable for analysing and measuring innovation in the H2020-MSCA-ITN projects (beyond the patents), we used the Qlik Sense platform to extract additional IR information for those projects.

**Figure 2.1.**
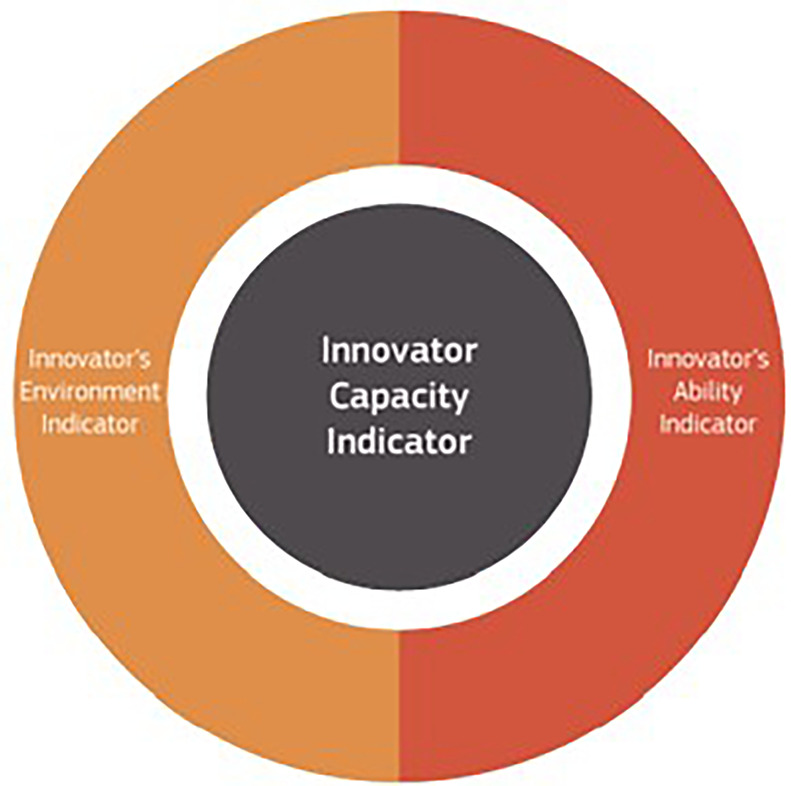
Innovator Capacity Indicator.

**Figure 2.2.**
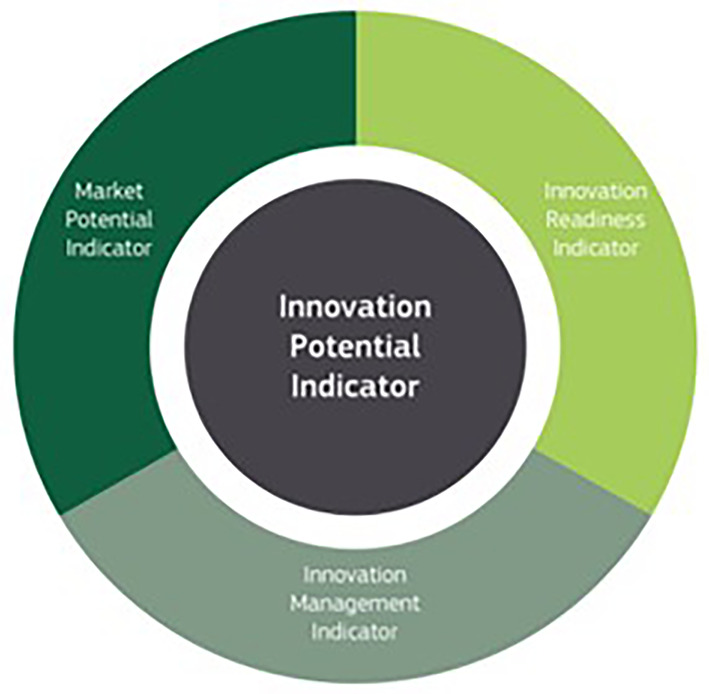
Innovator Potential Indicator.

We have further utilised the medium/high Innovator definition of IR and applied it to the MSCA selected projects. The goal of the IR Initiative is to identify high-potential innovations and Innovators in EU-funded research projects, seeking to direct project consortia on the proper steps to reach the market. Its goal is to maximise the results of public funds mobilised on research. Two indicators defined from the IR data ( Innovation Radar, 2018) have been selected as more suitable for our analysis: the **Innovation Potential Indicator** ( Figure 2.1), which measures the projects’ innovation development towards commercialisation within the framework programme, and the **Innovator Capacity Indicator** ( Figure 2.2), which captures the innovative capacity of the Innovators behind these innovations. The Innovation Potential Indicator incorporates three essential indicators in the innovation development process: the **Innovation readiness** (related to the technical maturity of the innovations e.g. prototyping, demonstration or testing activities or a feasibility study), the **Innovation management** (related to the project consortium capability and its commitment to bring innovations to the market, e.g. preparing a business plan or market study, defining the IPR ownership, securing capital investments, engaging end user), the **Market potential** (related to the demand and supply side of innovations e.g. evaluating the market conditions for successful commercialisation, assessing how products or services satisfy a market sector and a potential customer base or potential barriers from the supply side which could jeopardise the commercial exploitation of innovations). The Innovator Capacity Indicator includes two indicators that capture the capacity of Innovators in delivering successful innovations: the **Innovator’s ability** (related to the ability of organisations in developing innovations within the EU-funded activities e.g. the number of times organisations have been identified as key Innovators by the IR, the reviewers’ opinions about Innovators’ potential and independence in fulfilling the market potential of innovations), the **Innovator’s environment** (related to the performance of the project in terms of innovations, the overall conditions in the project consortium which an Innovator faces e.g. the commitment of relevant partners to exploiting innovations, the presence of organisations that are directly interested in exploiting the innovations, the positive ecosystem to facilitate the knowledge spill-overs between Innovators and their environment).

**Categories of Innovators**

The IR ( Innovation Radar, 2015) introduces three categories of Innovators: **Low, Medium and High Potential innovators**. The assignment to a category is based on mean and standard deviation (SD) values of the Innovator Capacity Indicator (ICI) for Innovators and uses percentile ranks. Ordering Innovators into three different categories based on percentile ranks allows their performance to be compared very clearly with the remaining innovations and Innovators in the sample. The percentile rank of an innovation or an Innovator is defined as the percentage of Innovators in the same sample that obtained a score at the same level or below that of the Innovator’s score. Typically, the assignment of inventors to three categories is based on the following rules:

$$\mathrm{Low}\;\text{Capacity Innovator}\;{\mathrm{ICI}}_{j}<{\mathrm{ICI}}_{\text{Mean}}-{\mathrm{ICI}}_{\mathrm{SD}}$$

$$\text{Medium Capacity Innovator}\;{\mathrm{ICI}}_{\text{Mean}}-{\mathrm{ICI}}_{\mathrm{SD}}\leq {\mathrm{ICI}}_{j}<{\mathrm{ICI}}_{\text{Mean}}+{\mathrm{ICI}}_{\mathrm{SD}}$$

$$\text{High Capacity Innovator}\;{\mathrm{ICI}}_{\text{Mean}}-{\mathrm{ICI}}_{\mathrm{SD}}\leq {\mathrm{ICI}}_{j}$$

where j is the observed ICI score of Innovator and Mean and SD are average and standard deviation of the ICI.

We extracted from the IR database the ICI assessment conducted on all H2020-MSCA-ITN projects selected for funding from the calls 2014-2015-2016 and 2017, and compared with all project ICI assessment of the rest of H2020 calls from 2014 to 2017.

3. The HRB Service 4 ^11^ (Module A: publishable reports of R&I project clusters analysis) as a proof of concept of the methodology by external experts.

We made use of the HRB Service 4 for the cross-validation of the - as they appear at the initial extraction - promising innovative results that were identified in the selected sample of projects. The main objectives of the HRB analysis were to:

i) contribute to the practical methodology for tracking innovation and innovative best practices in H2020-MSCA-ITN projects (via external independent experts as a proof of concept of the other components of the proposed methodology),
ii) collect and present the most relevant innovative elements and results (R&I results) of the selected population of H2020-MSCA-ITN projects,
iii) find commonalities among these ‘innovative’ projects to further derive best practises and common paths to successful research and innovation generation,
iv) showcase the excellence in research and innovation potential of the networks in specific scientific domain(s) by clustering/classifying the identified R&I outputs in such domains, where possible.

The HRB analysis was based on the extracted (from CORDA) portfolio of the 118 selected projects.

The desk analysis and processing of the collected data was assigned to six independent and competent experts contracted by the HRB service. These experts were provided with the necessary data from the 118 projects (innovations and patents), the IR reports/questionnaires (extracted by the IR platform) and with additional project-related documents, like reports, deliverables, description of work, etc. (extracted from the EC corporate databases), resulting in thousands of documents to be considered and analysed for the total of 118 projects. Samples of these input data can be found under “Dataset, metrics and indicators used for Horizon Results Booster analysis” ( Bitsios *et al.*, 2023b).

The HRB analysis first focused on the mapping of projects, based on country of operation, thematic priorities and objectives. This allowed the clustering of projects into a few scientific domains. The scientific domains were determined via an initial analysis of the fields of science provided in the CORDIS database. When this information was not sufficient to assign a given project to one specific scientific domain, further analysis was done on the project’s definition and outputs, to assign every project into one identified domain.

The second step was to map the nature of the organisations and entities that were engaged in the 118 projects. This mapping was based on criteria like country, activity type, number of participations, consortium size, ‘Innovators’ (based on the IR definition) as project participants and per scientific domain and LERU participation ^12^. This analysis provided key insights on the type and participation of the organisations in the 118 projects and led to very interesting conclusions on the mix of the so-called ‘Innovators’ among the sampled projects participants.

Finally, the definition of a set of indicators/criteria permitted the extensive analysis of quantitative aspects of the clustered projects’ best practices and innovative outputs, as well as their qualitative assessment ( HRB report, 2023).

The following sources were considered for the definition of the criteria and indicators for the processing of the clustered projects’ data:

- reported publications,
- international grants and/or prizes,
- patents, creation of spin-off, evidenced commercial exploitation of results,
- innovations identified by the IR service,
- innovation and novelties declared by the projects (not via the IR platform) in their reporting or in other project relevant documents,
- members of the League of European Research Universities (LERU) network,
- synergies with other H2020-MSCA-ITN projects and/or clustering with projects by similar research area of applications,
- interviewing the projects stakeholders to follow up on the most promising project outputs after project end.

As already mentioned under the ‘Limitations’, interviewing of the project stakeholders was not required in the end. The variety and amount of data that were collected by the rest of the sources/metrics were more than sufficient for the HRB analysis.

A summary of the set of criteria and indicators that were defined for the quantitative and qualitative assessment of the clustered projects’ best practices and innovative outputs, can be found under “Dataset, metrics and indicators used for Horizon Results Booster analysis” ( Bitsios *et al.*, 2023b).

4. The analysis of most frequently found terms (word clouds) which can be retrieved from the generated publications.

The dataset consisted of the H2020-MSCA-ITN projects from the call years 2014 to 2017. Similarly, the reported scientific publications for the same projects have been retrieved. We carried out a wordcount on the titles/keywords of the projects, as well as on the titles of scientific publications. The analysis was performed by scientific panels (Chemistry – CHE; Economics – ECO; Engineering and Information Sciences – ENG; Environment and Geosciences – ENV; Life Sciences – LIF; Mathematics – MAT; Physics – PHY; Social Sciences and Humanities – SOC), as the MSCA programme is bottom-up by nature and funds research in all fields of research. Due to their reduced sizes, the ECO and SOC panels were merged in a single panel (ECOSOC) and the same was done with the MAT and PHY panel (MATPHY). The analysis was carried out in R (R Core Team), with word sizes being proportional to their frequency and displayed in word clouds. Some correlation analysis was also performed between the results obtained among funded proposals and those among publications in order to verify if the trends at funded proposal stage (beginning of the project) can be a good predictor of the work done and reflected in the corresponding publications. The 50 most frequent and correlated words are displayed in each figure. Most of the generic terms, considered as noise, were removed from the analysis. The same analysis has also been performed on the subset of the 118 projects flagged for innovation.

5. The non-tangible innovation and best practices results which can be retrieved from the non-structured data in the different project reports and documents.

Several indicators and best practices were selected to analyse non-tangible innovation elements in the MSCA 2014-2017 ITN projects, such as social innovation, innovative training practices, innovative project management, long-lasting collaborations etc. The type of data that was missing and could not be retrieved automatically from our various databases was further defined. Most of the best practices are also linked to data that can only be collected manually from the projects’ reports and documents. In order to ease the checks and screening of projects, some categories of data were pre-defined for each selected indicator and best practice.

6. Questionnaires/surveys from the ESRs that participated in those projects.

As an indicator of non-tangible innovative results, the MSCA follow-up questionnaires immediately after the fellowship and two years after the end of the fellowship were examined. Respondents were asked for feedback regarding their training experience and career improvement. A total of 4431 respondents answered to the end of the fellowship questionnaire, while a total of 181 fellows responded to the questionnaire 2 years after the end of the fellowship. The questionnaires were anonymous; therefore, none of the replies can be traced back to a particular individual. The results presented in this document reflect the answers retrieved from these 4431 and 181 survey replies.

### Limitations

Past research on innovation ( OECD, European Union, 2018; Taques, López, Basso, Areal, 2020), already proposed a number of indicators for measuring innovation in different application domains. It must be noted that, for every identified indicator there are advantages and disadvantages on its use for measuring innovation. For example, patent databases can be useful when assessing innovation impact for research projects. On the other hand, while the use of questionnaires to track and measure innovative outputs allows identification of the type of innovation generated by a given organisation, it has certain drawbacks. It is prone to subjectivity (there is no control point for the information because the input is given by the same organisation claiming the innovation generation), not all innovation elements are reported due to information confidentiality concerns, and it is also prone to typos or clerical errors during the completing process ( Taques, López, Basso, Areal, 2020).

In the present study, another limitation concerns the collection of unstructured data, manually retrieved from various project reports and documents. Our methodology and introduction of the pre-defined categories aimed at reducing the subjectivity and the variability in the data analysis phase.

Regarding the population/sample of the projects that were analysed, we focused only on closed projects (from calls 2014-2017). For closed projects, normally the interim and final reporting has been conducted and the generated innovations and similar results have been encoded in the corporate systems, thus can be further analysed. Consequently, innovations and similar early results from on-going projects were not considered for this analysis.

The selection of the two main indicators (patents and innovations) for the identification of the sample of projects for the analysis may limit the representation of Social Sciences and Humanities (SSH) – related projects in the final selected sample. This can be considered as expected for two reasons: i) the generation of patents and similar innovation results are more likely to happen from Innovators that are industrial and/or commercial in their nature ( HRB report, 2023); and ii) the use and submission of patents and similar innovations, is traditionally reserved to domains of science and technology, like the STEM (Science, Technology, Engineering, Mathematics) and medicine/biomedicine/drugs disciplines ( HRB report, 2023; Reymond, 2020). However, relevant research has shown for example, that an in-depth analysis in patent sources can provide useful information on SSH topics beyond those of mere technological intelligence ( Reyomond, 2020).

As far as the HRB analysis is concerned, among the initially defined sources and metrics, interviews with selected project stakeholders were foreseen. However, interviewing project stakeholders was considered time-consuming while at the same time, the abundance of data collected by the rest of the defined sources made clear that there was no real need for interviews to complement the collected data.

Certain limitations and assumptions apply to the information and outputs presented in this study since the CORDA database and IR database are not wholly consistent nor harmonised across projects’ data. As such, the amount and granularity of information available exhibited variability.

## Results and main findings

### Analysis on patents and innovation outputs (CORDA and Qlik Sense)

As mentioned earlier, a focus on the 2014-2017 calls was considered more relevant. The H2020-MSCA-ITN 2014-2017 portfolio comprises 550 executed (on-going or closed) projects (see Figure 1.b.1).

**Figure 1.b.1.**
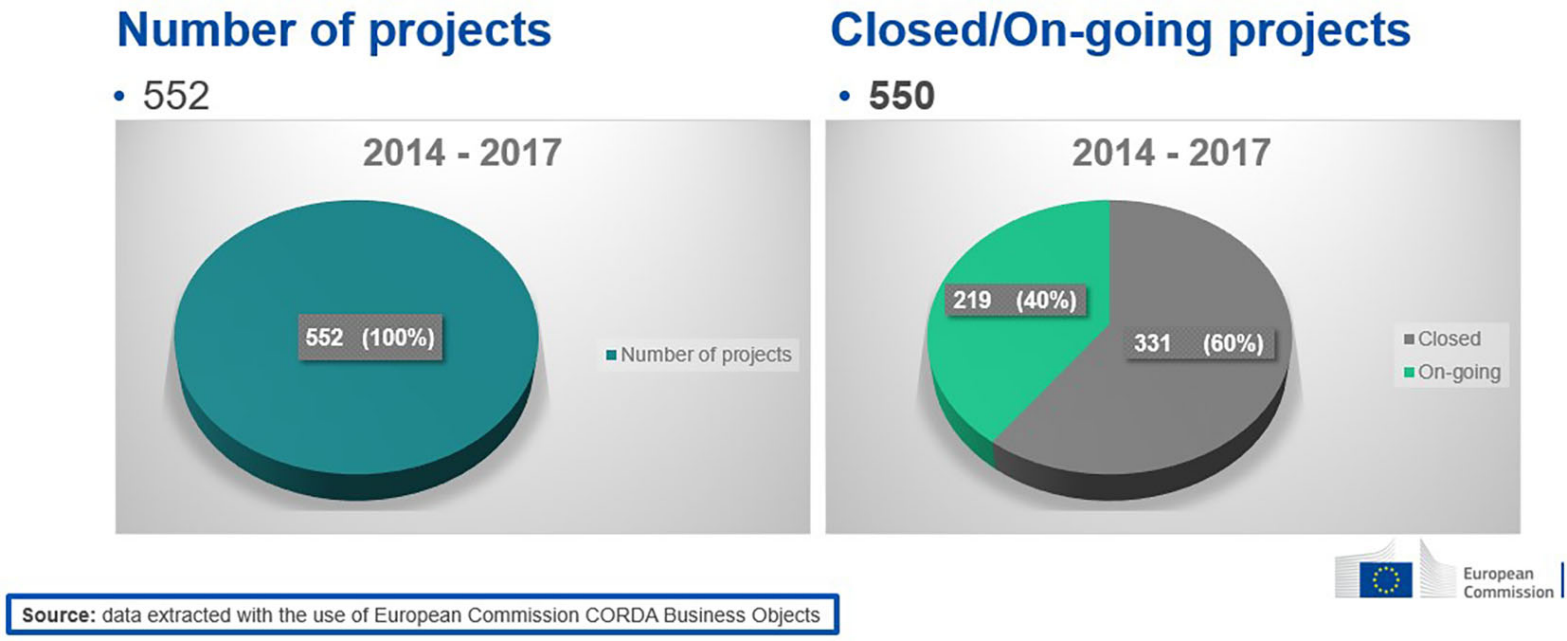
Number of H2020-MSCA-ITN projects under 2014-2017 calls (the 552 population includes 2 terminated/rejected projects).

From those projects, the vast majority (77%) were ETNs, while the rest were EIDs (16%) and EJDs (7%) (see Figure 1.b.2).

**Figure 1.b.2.**
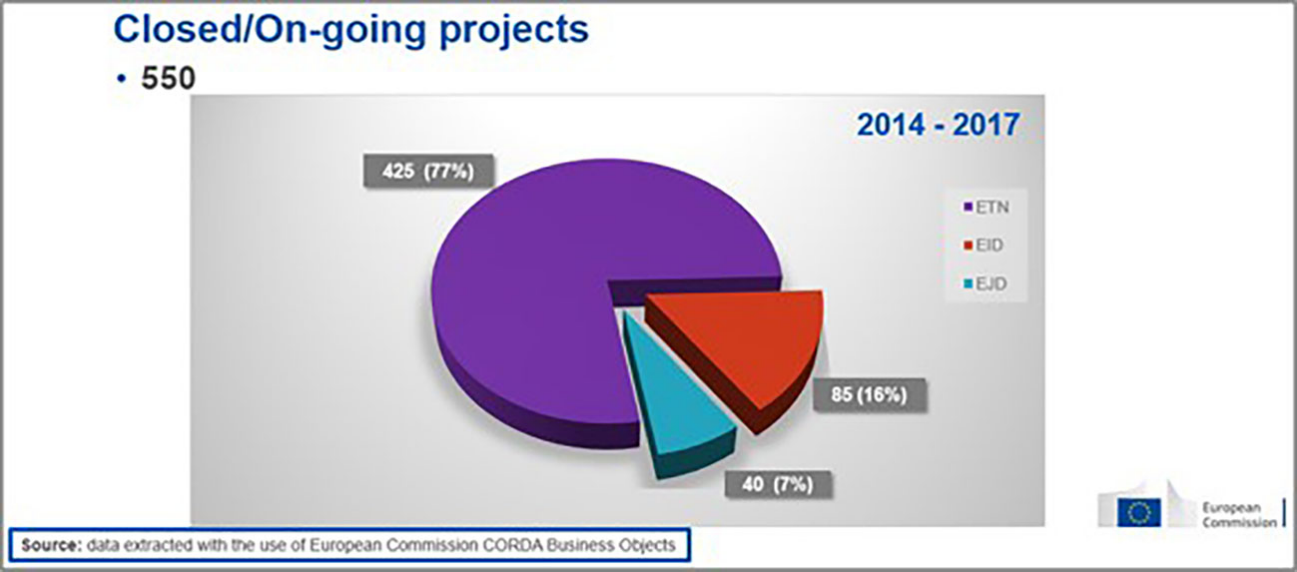
Mode distribution (ETN-EID-EJD) under 2014-2017 calls.

The distribution of modes in the 2014-2017 projects portfolio is in coherence with the distribution of modes in the total H2020-MSCA-ITN projects population from all calls 2014-2020 (78% were ETNs, 15% were EIDs and 7% were EJDs). Moreover, an analysis of the 2014-2017 population of projects based on the H2020 KPIs, like the number of publications and patents per 10 million euros funding, revealed that, in terms of publications, H2020-MSCA-ITN exceeded the established target. As for patents, although this indicator is not applicable to MSCA under the Excellence pillar of H2020, H2020-MSCA-ITN 2014-2017 projects showed a good performance in terms of the ratio of generated patents (see Figure 1.b.3).

**Figure 1.b.3.**
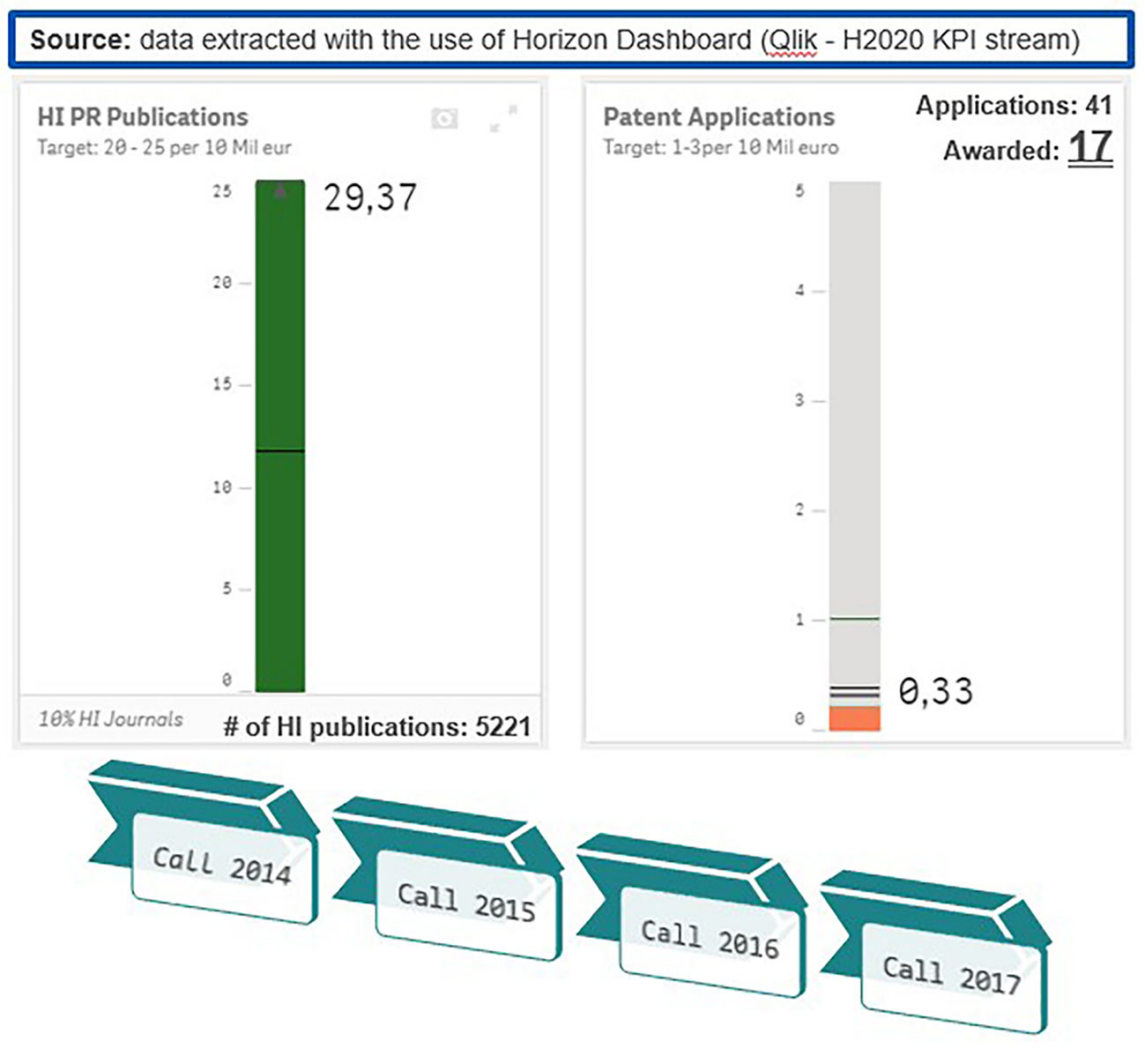
Analysis of performance of H2020-MSCA-ITN 2014-2017 projects based on the H2020 KPIs (generated publications and patents per 10 million euros funding).

From the extracted data, a filtering of the projects was done based on declared innovation(s) and/or patent(s). Out of the 550 analysed projects, 432 declared no generated innovation or patent, while the remaining 118 projects declared the generation of at least one innovation output or patent or both. Since the focus of this study was to measure the innovative dimension under H2020-MSCA-ITN, a deeper analysis was performed on the 118 projects with only innovation or patent output or with both. The analysis showed that out of those 118 projects which declared the generation of at least one innovation or patent output, 93 projects declared at least one generated innovation and no patents, 17 projects declared at least one patent and no innovation, and 8 projects declared at least one innovation and one patent (see Figure 1.b.4). Notably ETN mode contributes to all types of innovation outputs (only inno, only patent, inno and patent).

**Figure 1.b.4.**
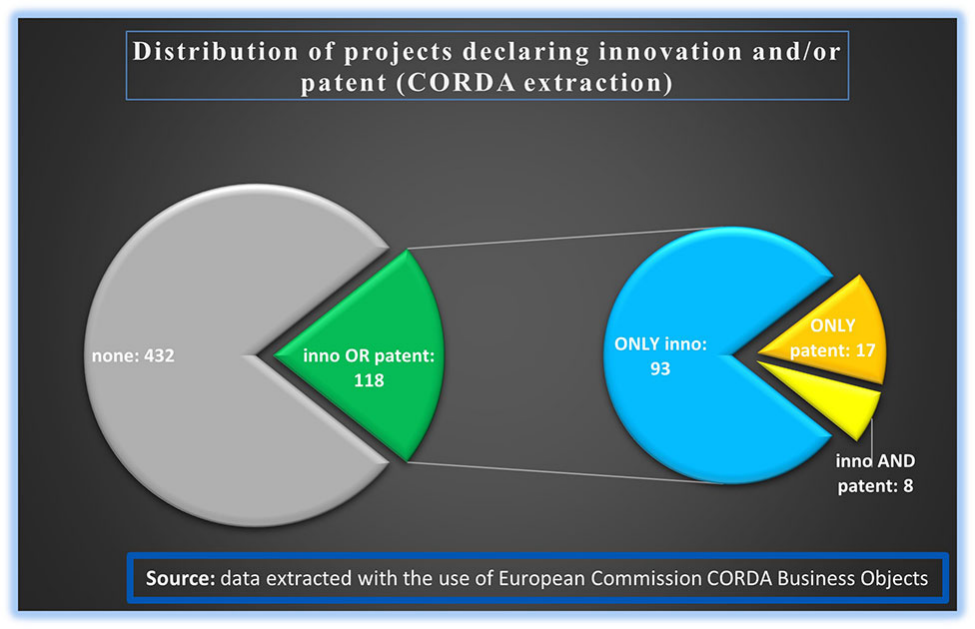
H2020-MSCA-ITN projects under 2014-2017 calls that generated at least one innovation output and/or at least one patent and additional breakdown of these projects (118) based on the occurrence of only innovation (93) or only patent (17) or both (8).

We further examined the mode distribution in the 118 projects with the sole purpose of verifying that they can be considered as a good representative sample of the total 2014-2017 population of projects. Indeed, out of these 118 projects, 92 projects (~78%) were ETN, 20 projects (~17%) were EID and the rest, 6 projects (~5%), were EJD (see Figure 1.b.5). This mode distribution among the 118 selected projects is similar and comparable to the mode distribution among the total number of 2014-2017 calls’ projects (see Figure 1.b.2).

**Figure 1.b.5.**
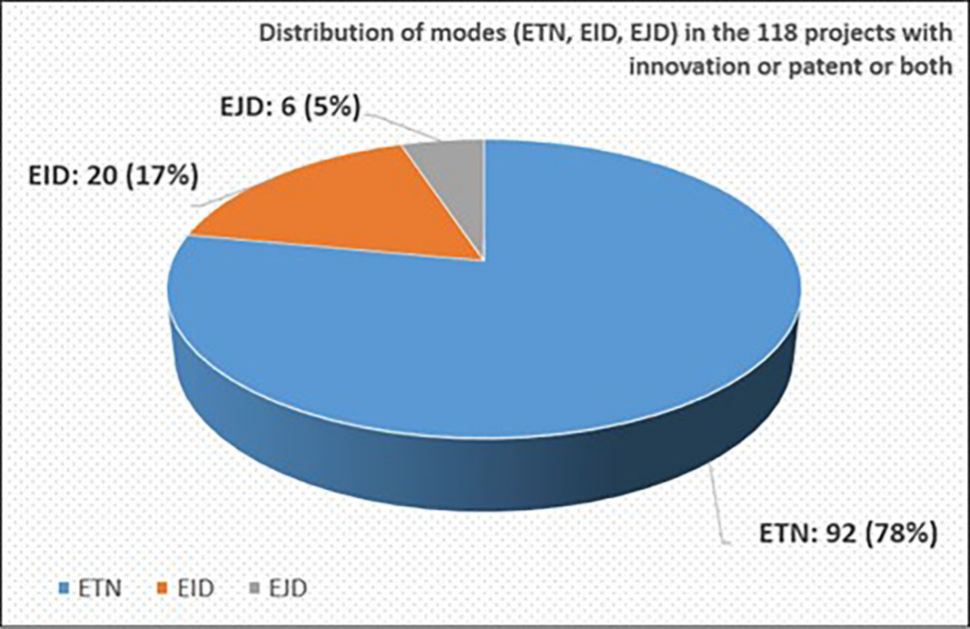
Mode distribution (ETN-EID-EJD) in the 118 projects with innovative dimension.

In terms of patent performance based on the project mode, the analysis of 118 projects showed that EIDs included in this sample, tend to report proportionally more patents per EID project (~1 patent/EID project) than ETNs of the same sample (see Figure 1.b.6). This is consistent with the expectation that EIDs which are more industry-oriented, prioritise the generation of patents.

**Figure 1.b.6.**
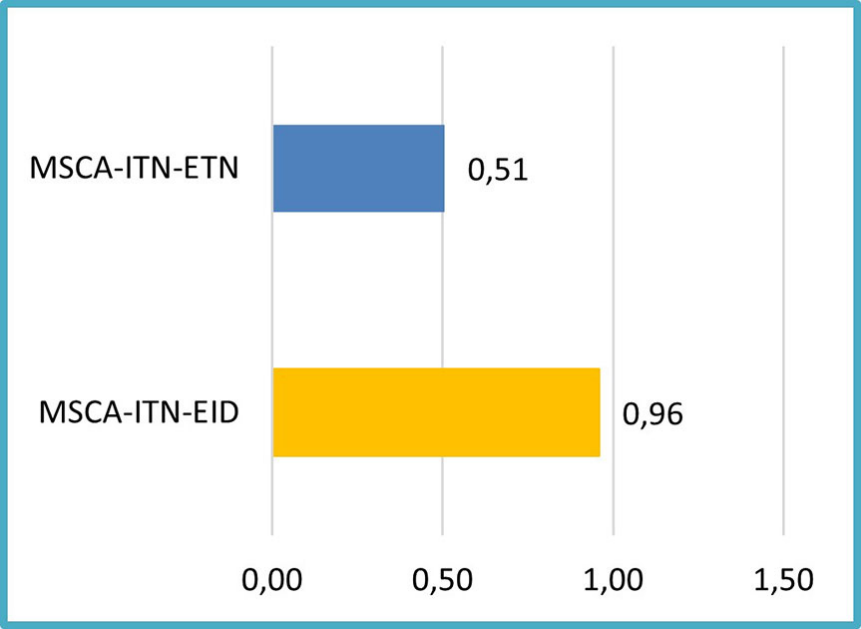
Number of patents per flagged project for innovation (118 projects) (normalized project relative size).

Based on the findings of this first component of the methodology, we are already in the position to consider the selected 118 projects as a very good and representative sample to measure, analyse and draw conclusions on the innovative aspects of the H2020-MSCA-ITN projects. As already mentioned, additional components were considered in the methodology to further validate the credibility of the selected sample, and these are elaborated in the following paragraphs.

### The Innovation Radar indicators

a. Innovation Radar analysis

In order to further confirm that the selection of the 118 projects is the best and the most promising candidate for analysing and measuring innovation in the H2020-MSCA-ITN projects (beyond the patents), we extracted additional IR information for those projects (see Figure 2.b.1).

**Figure 2.b.1.**
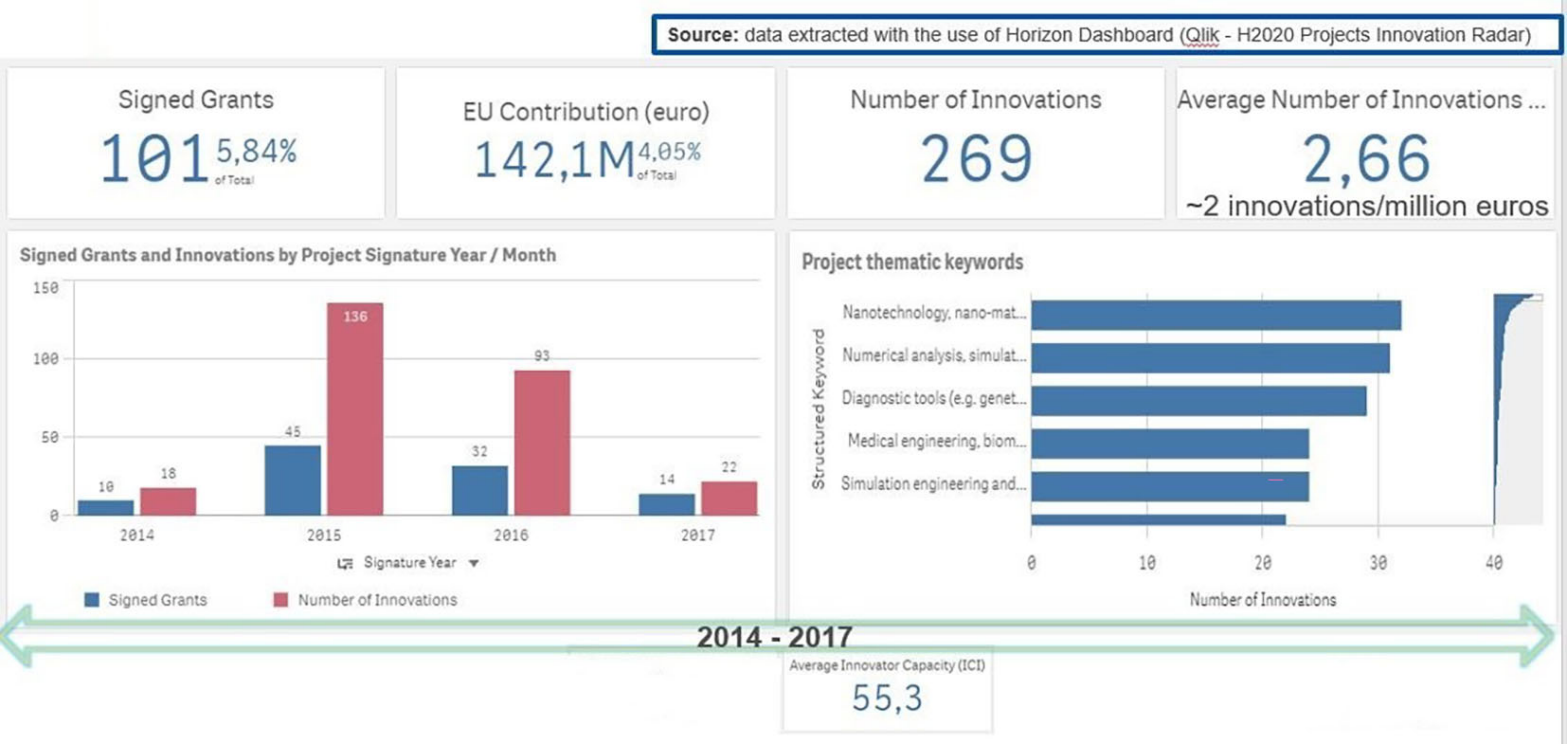
Data on generated innovation for the 101 projects (out of the selected 118 projects).

It is to be noted here that the extracted information from Qlik Sense platform on generated innovation for the selected 118 projects, is actually restricted to 101 projects for the H2020-MSCA-ITN from 2014-2017 calls. This is logical, since 17 projects out of the 118 selected projects (see Figure 1.b.4) have reported only the generation of patents and no innovation outputs (at least in the sense of innovation outputs that are captured via the IR platform).

b. Innovator Capacity assessment, comparison between MSCA and the rest of H2020

We have extracted from the two IR platform databases (up to April 2018 and from May 2018 onwards) the ICI assessments conducted on those 101 projects coming from H2020-MSCA-ITN, calls 2014-2015-2016-2017 and all other H2020 projects from the same period (calls 2014 to 2017).

We calculated, according to the IR methodology, the average ICI and standard deviation of the H2020 selected population and MSCA one. These values computed as Average +/- Standard Deviation, determine the ICI values that define the low, medium, high Innovator categories in both population under analysis. These values and the percentage of the organizations in each category are reported and compared in Table 2.b.1.

**Table 2.b.1.** Percentage of organizations in each innovator category by H2020 and MSCA-ITN populations.

HIGH INNOVATORS	%	ICI innovator category values
high innovators in H2020 vs total inn.	15.0	>68.6
high innovators in MSCA ITN vs total inn.	14.2	>67.4
**MEDIUM TO HIGH INNOVATORS**		
medium to high innovators in H2020 vs, total inn.	82.9	>43.1
medium to high innovators in MSCA ITN vs total inn.	83.6	>40.3
**MEDIUM INNOVATORS**		
medium innovators in H2020 vs total inn	67.9	>43.1 <68.6
medium innovators in MSCA ITN vs total inn.	69.0	>40.3 <67.4

From the comparison, it is clear that the two populations performed equally in terms of Innovators’ distribution per innovation category, showing similar percentage of medium to high Innovators vs the total Innovators. We have to underline that H2020-MSCA-ITN, being under the excellence science pillar, involves young researchers with less than four years research experience employed in training networks mainly focused on providing the necessary knowledge and expertise to boost the young researchers’ career prospects within an innovation ecosystem. So the fact that H2020-MSCA-ITN shows similar capacity to generate innovation as of H2020 programmes, whose innovation outcome are coming from Research and Innovation Actions (RIA) and Innovation Actions (IA) projects where the main players are senior researchers, scientists employed in academic and non-academic organisations, is a truly remarkable achievement of the H2020-MSCA-ITN actions.

c. Follow-up of HRB study on H2020 and H2020-MSCA-ITN common Innovator organisation type analysis

Following the results of the HRB analysis, conducted on the 118 projects, we have developed on the ‘Innovator’ concept, based on the IR definition for the 101 projects assessed by IR out of the 118. According to the HRB study:

➢ **When it comes to beneficiaries, “success breeds success”** in that some are significantly more active than others in project participation, which in turn builds upon their capability to be further innovative and successful. Beneficiaries that are in nature industrial, such as companies working in pharmacological development, and identified as Innovators are those being the most innovative. Therefore, encouraging cooperation with coordinating Innovators can boost project success, while new ways of expertise sharing will support diversity and inclusiveness, as well as success, amongst consortia.

We have further investigated this important result, analysing the organisation type of those Innovators present in 101 out of the 118 projects studied by HRB, in comparison with the entire population of Innovators in H2020 projects originated from the same calls of reference. Out of 265 organisations considered as Innovators in H2020-MSCA-ITN, 240 are also Innovators in H2020. We have used the ICI values of H2020 to define the innovator categories (low, medium and high) of those common Innovators per organisation type; academic (HES, RES, PUB) ^13^ and non-academic (PRC) differentiating within the non-academic sector group between SME and non-SME. The distribution of organisation type is shown in the following Table 2.b.2.

**Table 2.b.2.** Distribution of organization type by category of innovators.

Innovators categories	SME	PRC	HES/RES/PUB	TOT
high innovator in MSCA-ITN (above 68.6)	9	5	18	32
medium innovator in MSCA-ITN (>43 ICI)	34	41	112	187
lower innovators in MSCA-ITN and H2020(<43.1)	2	2	17	21
All innovator in MSCA-ITN and in H2020	45	93	102	240

The pie chart analysis per Innovator category (see Figures 2.b.1a, 1b, 1c) shows that the higher the Innovators’ ICI of one organisation, the higher the concentration of SME within a certain Innovator category, confirming once more the finding of the HRB study. In the high Innovator band, we found 28% of SME while in the medium one 18%, and in the lower one, only 9%, as shown in the relevant Figures. This is not surprising since the high Innovator character is linked to high technology readiness level, which is an essential element pursued by organisations who want to be closer to the market valorisation and thus adapt their exploitation plan according to the projects they are in. Non-academic organisations and particularly SMEs are indeed more interested in high innovation that brings them closer to market valorisation. In fact, the lower the ICI, the higher is the percentage of non-profit organisations, namely universities, research and public bodies.

**Figure 2.b.1a.**
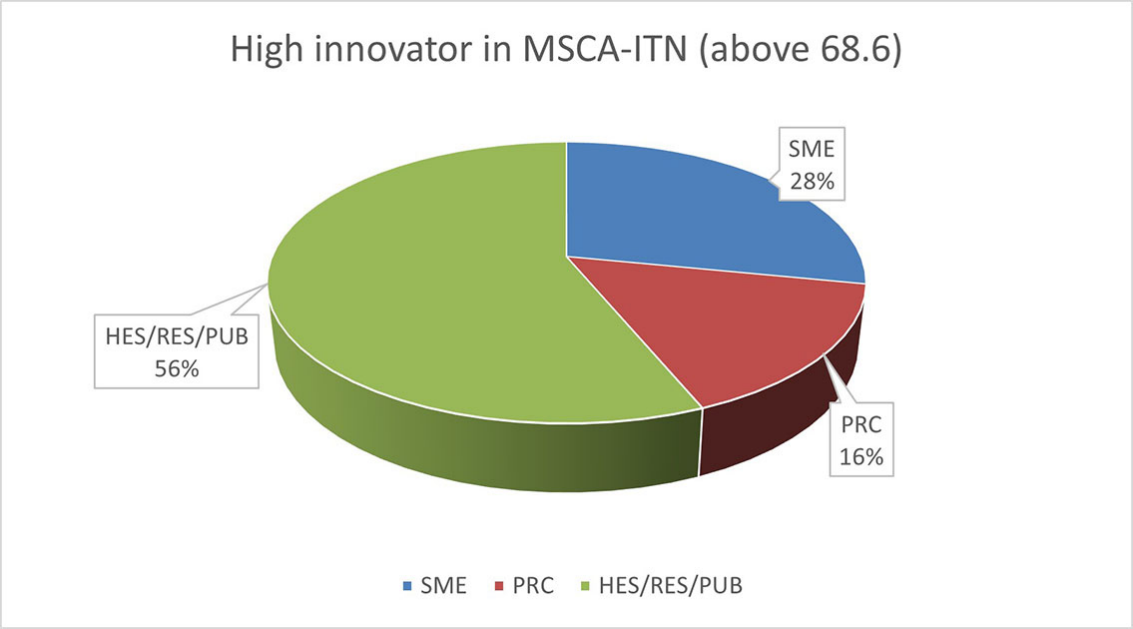
High Innovator in MSCA-ITN per organisation type.

**Figure 2.b.1b.**
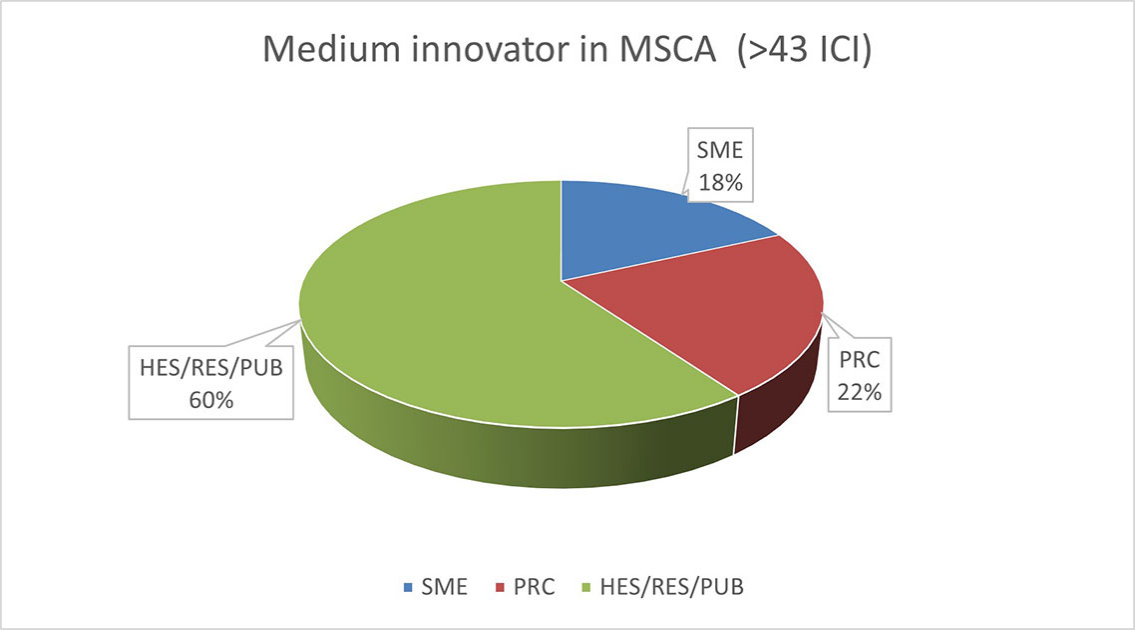
Medium Innovator in MSCA-ITN per organisation type.

**Figure 2.b.1c.**
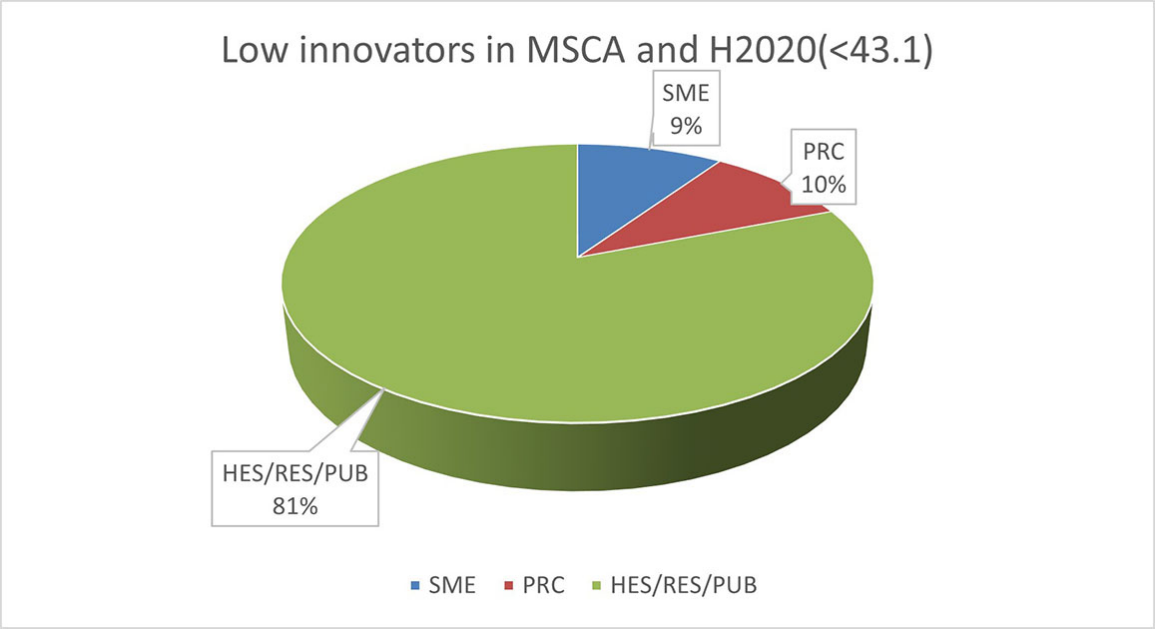
Low Innovator in MSCA-ITN per organisation type.

To highlight this finding, out of the 13 organisations defined as high Innovators in both H2020-MSCA-ITN and H2020, 9 (69%) are SMEs as shown in Figure 2.b.2. This once more confirmed the interest of the non-academic sector (mainly SME) in being at forefront of the innovation.

**Figure 2.b.2.**
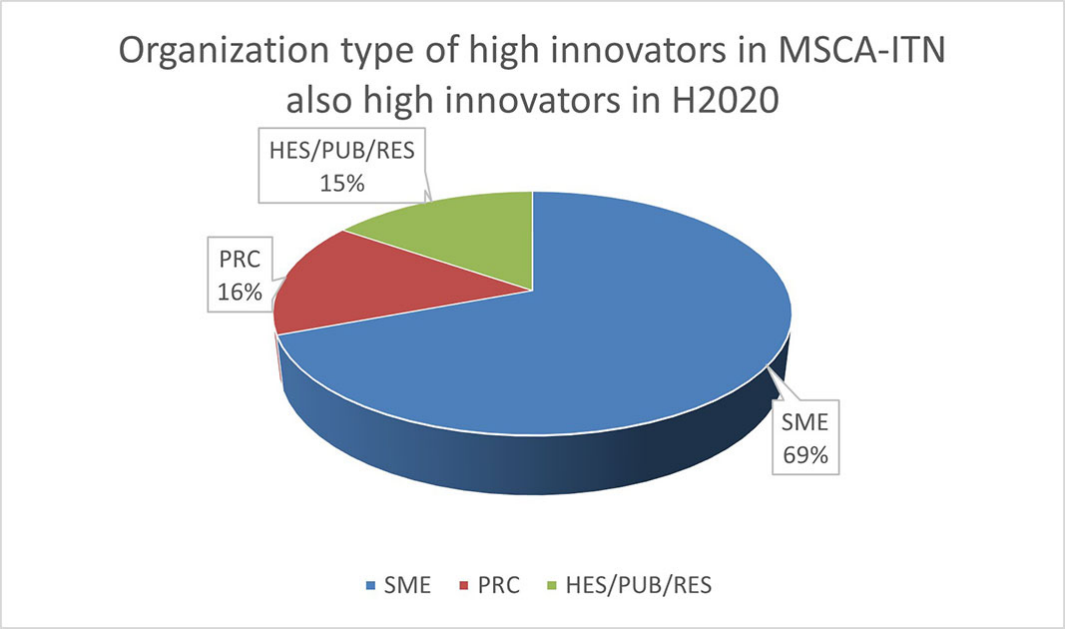
Organisation type of high Innovators in MSCA-ITN which are also high innovators in H2020.

### Horizon Results Booster (HRB) analysis (proof of concept)

The HRB analysis ( HRB report, 2023) generated a number of very interesting results for the portfolio of 118 projects funded by the H2020-MSCA-ITN Programme taking into account four consecutives calls 2014-2015-2016-2017. This section summarises the key results and findings.

The projects and their outputs were clustered into 15 scientific domains. The clustering of the projects into the scientific domains allowed more efficient identification of commonalities amongst the projects and the comparison of the innovative elements between these scientific domains. These domains and the distribution of projects (in total 118 projects) can be seen in Figure 3.b.1.

**Figure 3.b.1.**
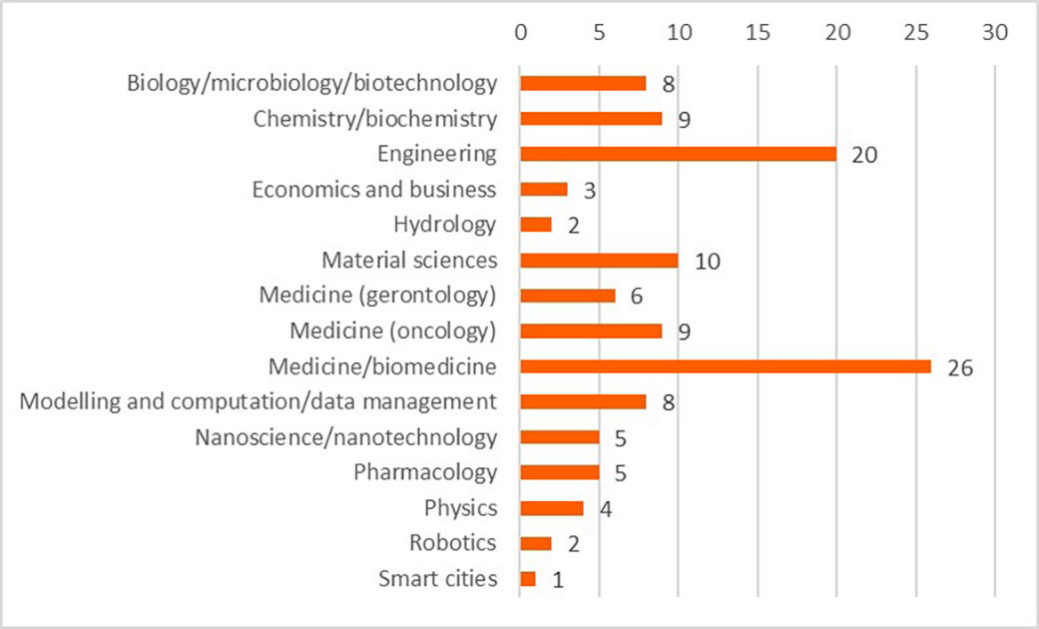
Scientific domains and distribution of clustered projects (for the 118 projects).

The mapping exercise of the organisations that were engaged in the 118 projects revealed among others very promising results in terms of the innovation potential of the involved organisations and the frequency of their participation in the H2020-MSCA-ITN action. The results indicated that 628 organisations participated in the analysed projects and while most of the organisations (almost 73%) were involved in only one analysed project, more than one-fourth (~27%) of the organisations demonstrated more active engagement. In terms of countries’ representation among the organisations involved in the analysed projects, 475 (out of the 628 in total, almost 76%) are located in the EU-14 countries ^14^, 122 in non-EU (~19%), and only 31 (almost 5%) in the EU-13 countries ^15^. From the engaged organisations, 60% of the project participants represent higher education institutions and research organisations (276 and 98), while 238 (38%) are companies from the private sector. The analysis showed that a project consortium is typically built of eight organisations and on average it covers four higher education institutions, three private enterprises, and one research organisation. Of the 171 organisations that joined more than one project, 100 fall under the category of ‘Innovators’ and most of the projects had at least one Innovator in their consortium. From the total number of ‘Innovators’ identified in the 118 projects, 77% are located in the EU-14 countries, 3% in the EU-13 countries and 20% in non-EU countries. Similarly, as for the project participants in general, 60% of the ‘Innovators’ represent higher education institutions and research organisations and 39% are companies from the private sector. On average, four ‘Innovators’ participated in one project and in most projects, at least one ‘Innovator’ was involved. Moreover, and regarding ‘Innovators’ amongst the defined (15) scientific domains, they are well represented as participating organisations across all scientific domains. Interestingly, the mapping exercise of the involved organisations showed that the members of the League of European Research Universities (LERU) network are sufficiently represented in most scientific domains (see Figures 3.b.2, b.3, b.4, b.5, b.6, b.7).

**Figure 3.b.2.**
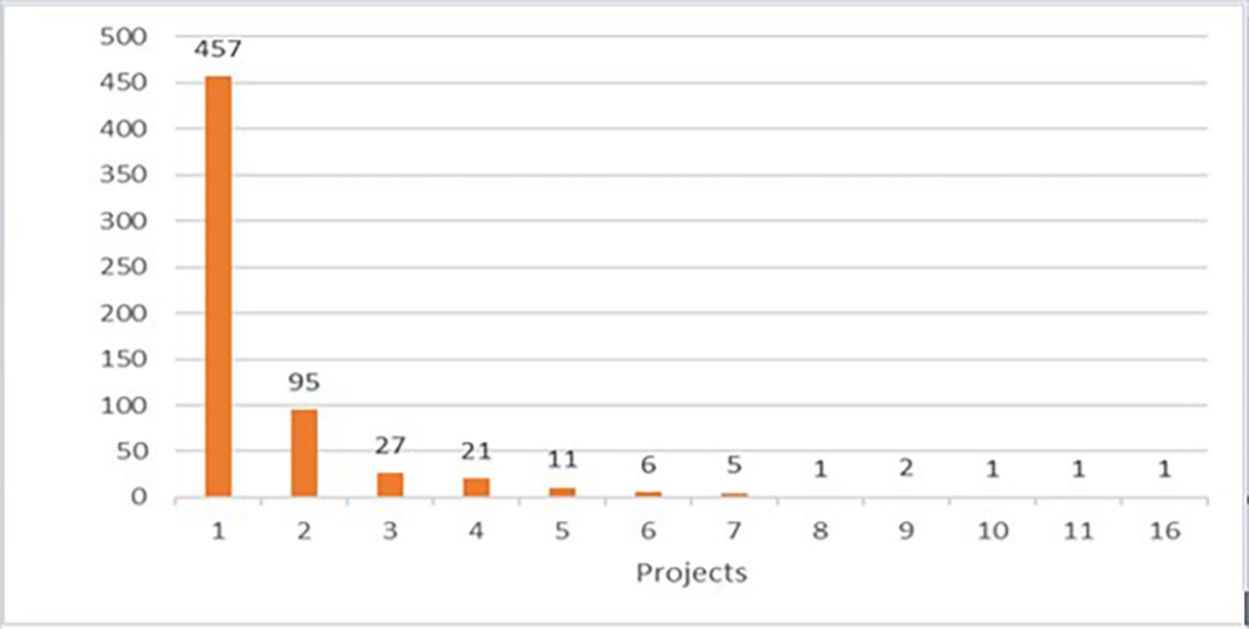
Participants’ engagement in the analysed projects.

**Figure 3.b.3.**
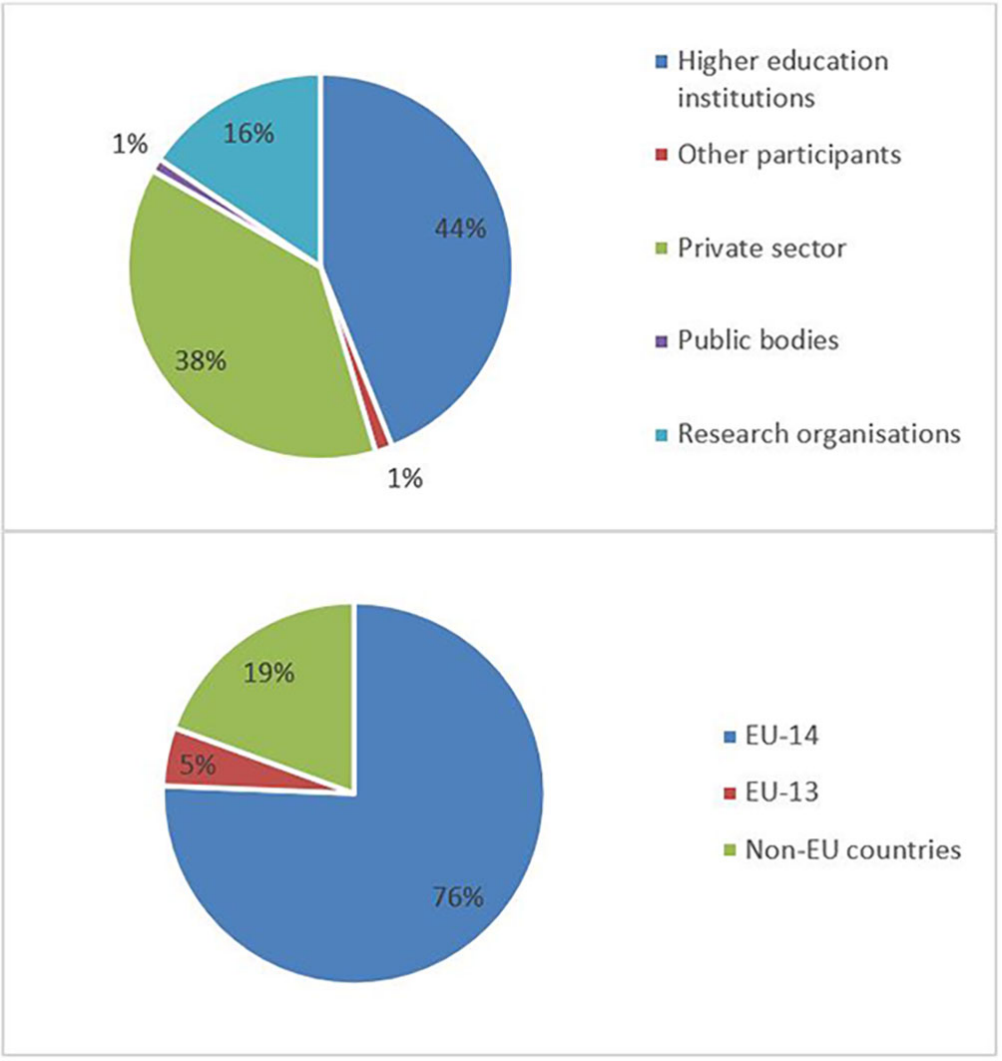
Participating organisations per type of activity and country.

**Figure 3.b.4.**
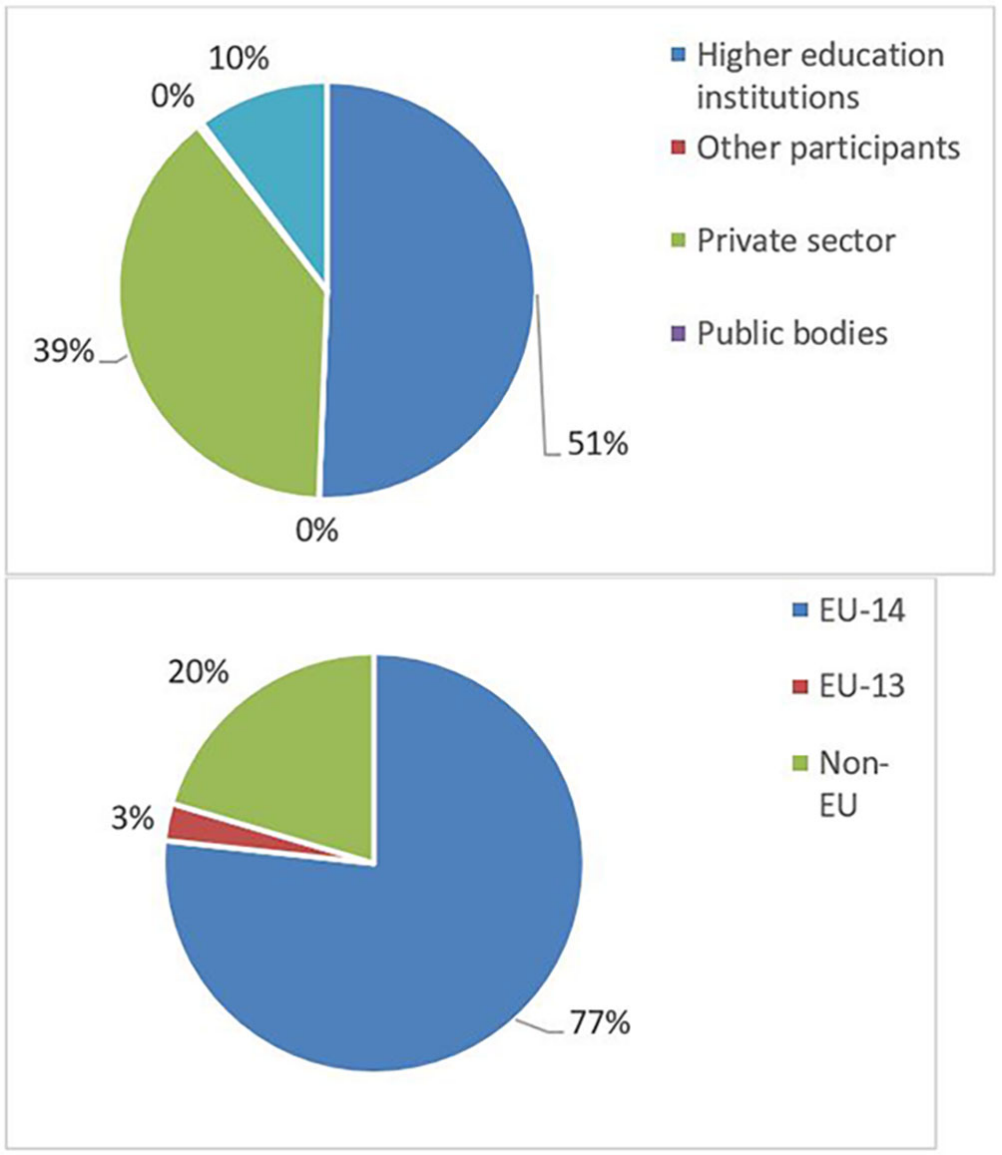
‘Innovators’ per type of activity and country.

**Figure 3.b.5.**
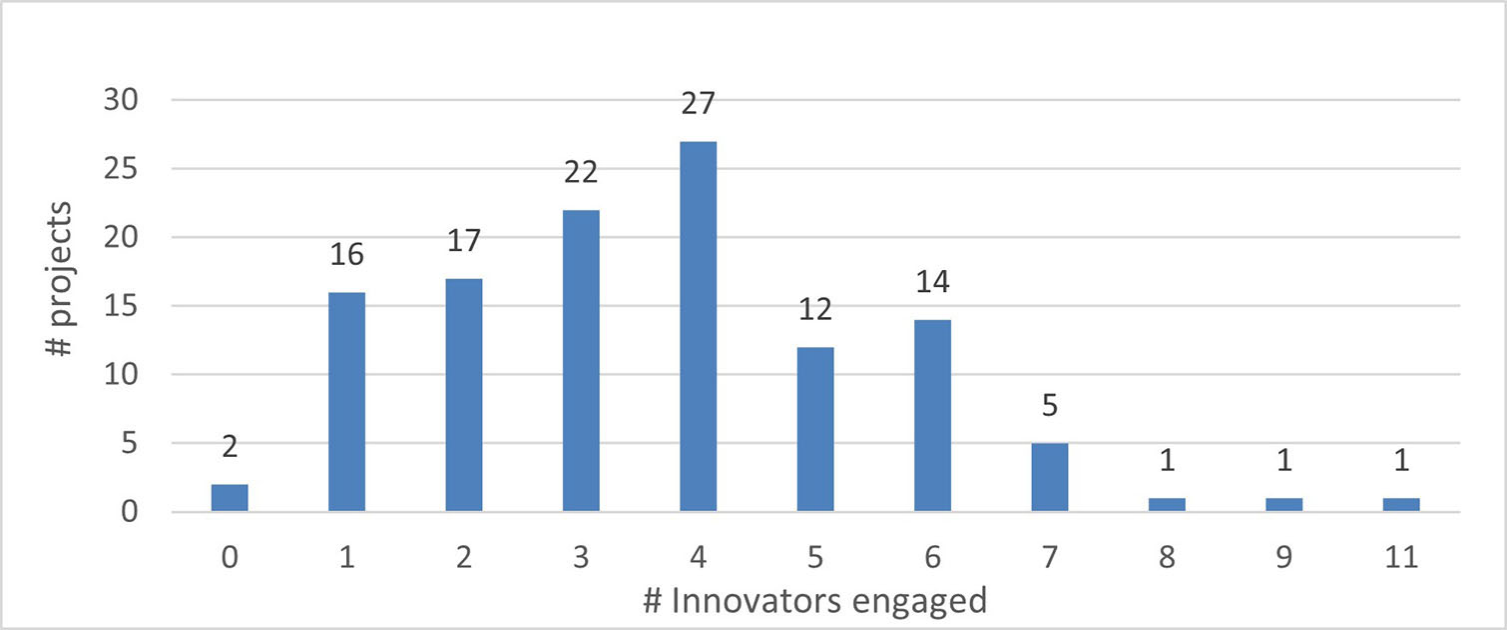
The representation of ‘Innovators’ in the projects. For example, in 27 projects (out of the 118 projects) 4 ‘Innovators’ were part of the project participants. Only in 2 projects (out of the 118) no ‘Innovator’ was part of the project participants.

**Figure 3.b.6.**
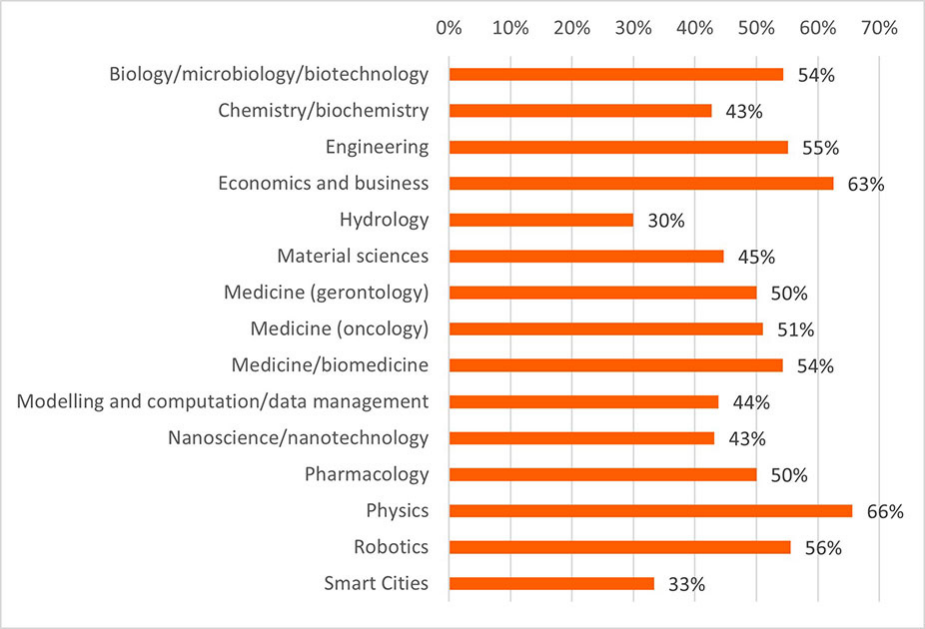
Share of innovators of all participants per scientific domain.

**Figure 3.b.7.**
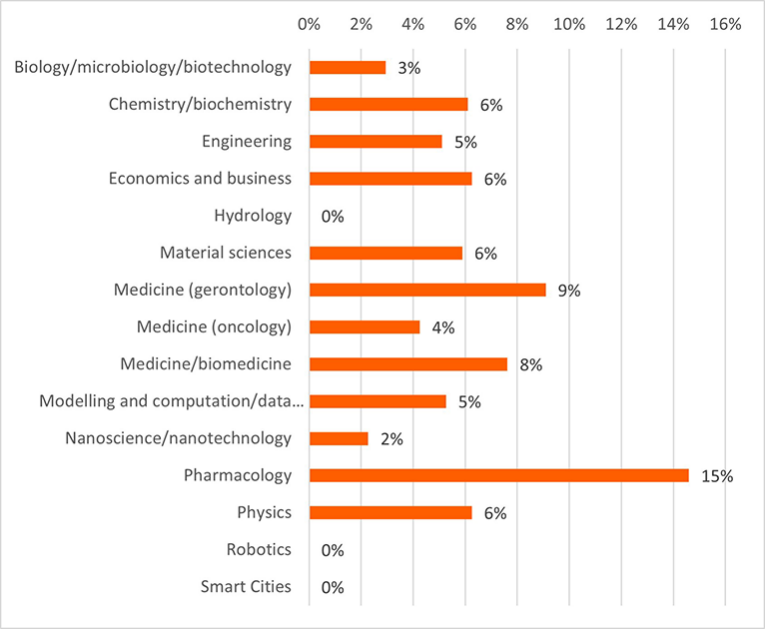
Share of LERU members of all participants per scientific domain.

Concerning the results for innovative outputs (i.e. patents, publications, innovations from the IR service), the analysis revealed that certain scientific domains are more prone to generating patentable outputs, possibly due to their results having competitive market applications. In terms of publications, all scientific domains have significant output in terms of peer-reviewed articles. Some are more prolific in this regard than others. This could suggest possible differences between the domains regarding certain factors, for example the novelty of the research area, the availability or feasibility of research and experimentation, or the internal dynamics of domain research communities. Finally, the innovation output performance as an average number of innovations was identified and was presented, per scientific domain, which can suggest the innovation potential of a domain based on current research challenges etc. (see Figures 3.b.8, b.9, b.10).

**Figure 3.b.8.**
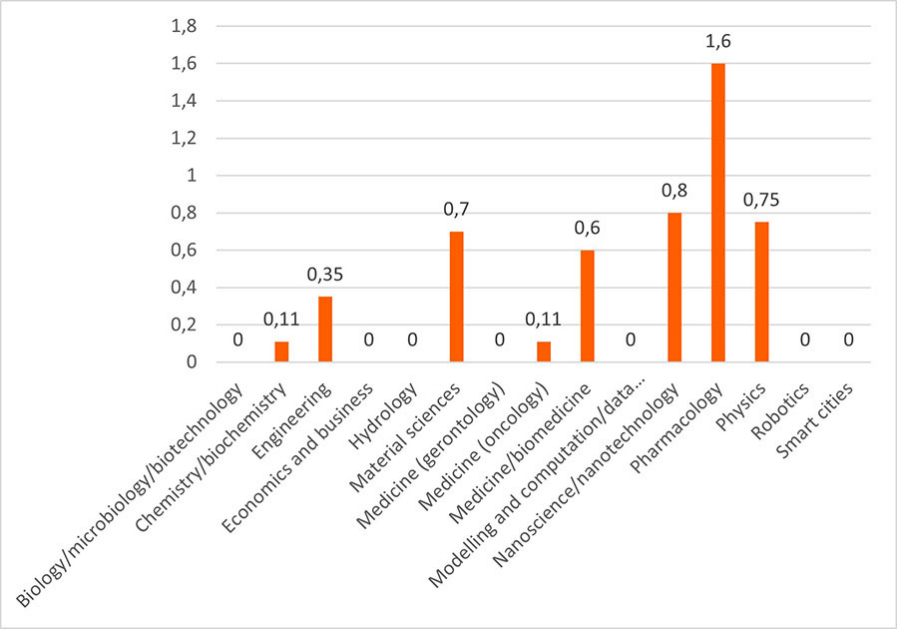
Average number of patents per scientific domain.

**Figure 3.b.9.**
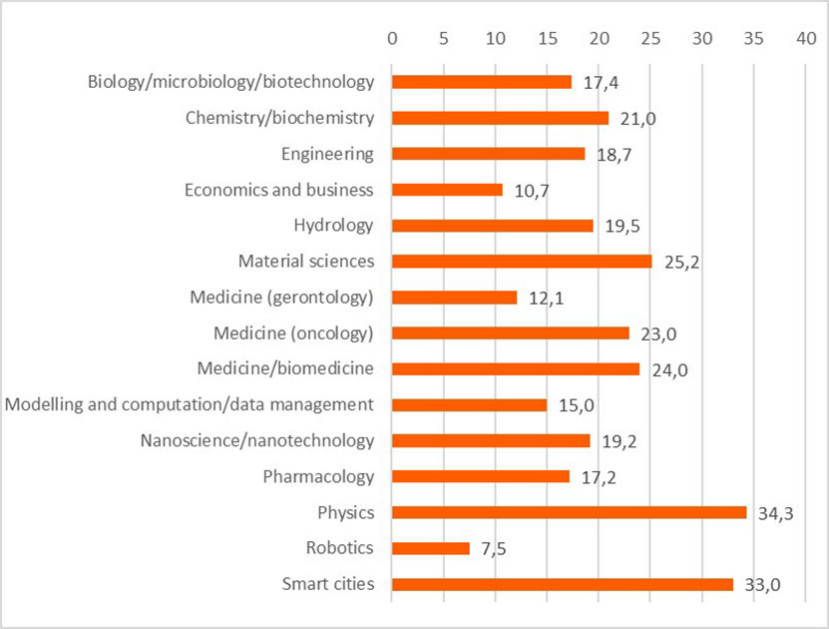
Average number of peer-reviewed articles per scientific domain.

**Figure 3.b.10.**
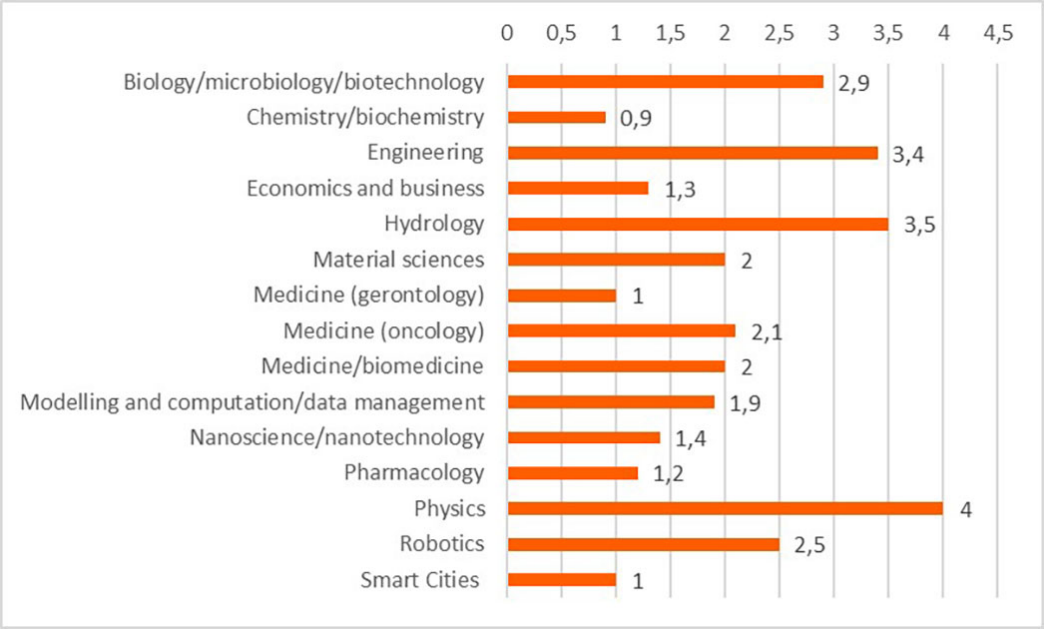
Average number of innovations identified by the Innovation Radar per scientific domain.

Finally, in terms of the capability of the analysed projects and/or their results, to be transferred elsewhere, to be applied elsewhere or to be able to engage in synergies with other projects (so to be considered as innovative/best practices), the HRB analysis provided remarkably interesting results. For example, projects with large numbers of events, particularly conferences, were found in almost every scientific domain, which can suggest the effort for public outreach of the projects (integral part of the H2020-MSCA-ITN programme) in order to ‘transfer new knowledge to scholarly communities and communicate it to society’, in line with good practice elements for doctoral training ( LERU advice paper, 2014). Regarding performed secondments (also an integral part of the H2020-MSCA-ITN action), the qualitative feedback provided by the participating projects in the IR questionnaires, indicated that the projects consider secondments to be important and would like them to be further supported and enabled. These elements can be assumed as part of the non-tangible innovation that can be observed in the H2020-MSCA-ITN projects, where best practices, such as the mobility of the recruited researchers (for example via the secondments), the participation in wide training events, or the cross-sectoral cooperation (via the exchange of researchers in academic and non-academic participants) nurture the co-creation of novel solutions (which are then reported as innovation outputs). The diffusion and transfer of knowledge, and the cross-sectoral cooperation, have been put in place to stimulate innovation creation.

The detailed results and interesting findings of the full HRB analysis can be found in the published HRB report ( HRB report, 2023), which was generated in the context of this study. The HRB analysis led to key conclusions and concrete links to policy-feedback suggestions and recommendations, which are presented in the ‘Conclusions and main recommendations’ section.

### Analysis of most frequently found terms (words clouds) which can be retrieved from the generated publications

The purpose of this analysis was to assess, using a visual representation, what the most frequent words and topics in H2020-MSCA-ITN projects are and if some trends identified at the project level (intended research) can be a good predictor of the effective research performed (outputs such as scientific publications).

It must be noted here that, as it can be observed in Figure 1.b.3 (related to the extraction of the calls 2014-2017 projects), the performance of the H2020-MSCA-ITN programme in terms of publications output was above the average in comparison to the whole H2020 programme. The beneficiaries of those 550 projects from the H2020-MSCA-ITN from 2014-2017 calls, have reported (as of December 2021) a total of 8883 scientific publications ( Table 4.1).

**Table 4.1.** Distribution of MSCA ITN selected projects per call year and respective reported publications.

Call year	Nb projects	Nb reported publications	Nb publ./proj.
2014	141	2453	17,4
2015	136	2503	18,8
2016	133	2607	19,6
2017	140	1320	9,4
**Total**	**550**	**8883**	**16,2**

ENG and LIF panels represent almost 60% of the total ( Table 4.2). When looking at the scientific productivity of H2020-MSCA-ITN projects, the two largest panels (ENG and LIF) have the highest shares of publications in absolute terms, but the Chemistry (CHE) and Mathematics/Physics (MATPHY) panels are more productive in relative terms (with an average of 24,3 publications/project for MATPHY).

**Table 4.2.** Distribution of MSCA ITN projects per panel and respective reported publications.

Panel	Nb projects	Nb reported publications	Nb publ./proj.
CHE	69	1185	17,2
ECOSOC	52	566	10,9
ENG	165	2514	15,2
ENV	67	1029	15,4
LIF	151	2471	16,4
MATPHY	46	1118	24,3
**Total**	**550**	**8883**	**16,2**

**Figure 4.1.**
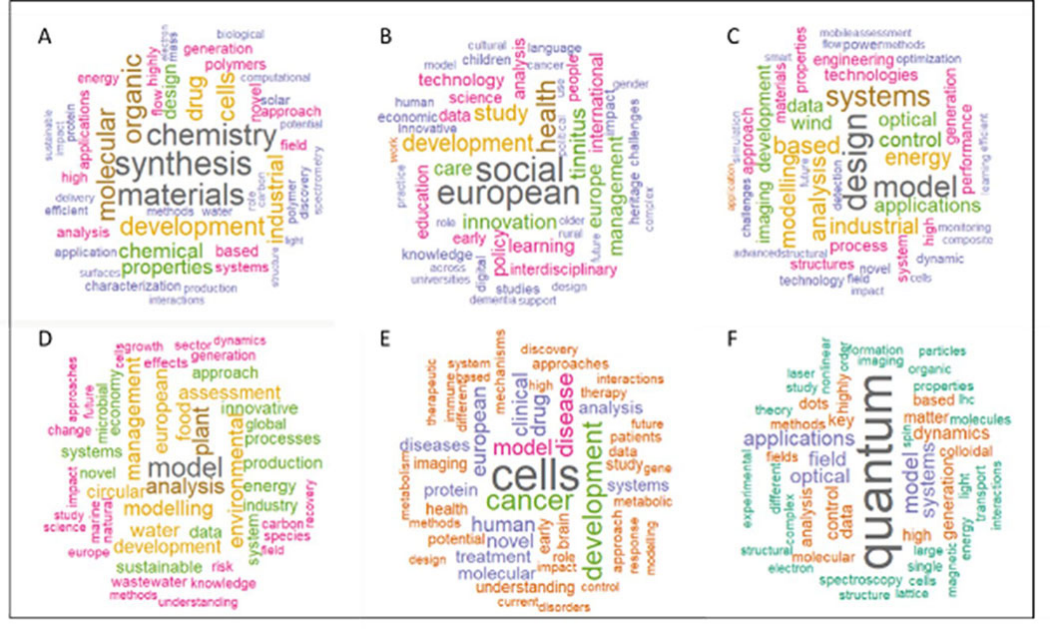
Correlated words frequencies in projects’ titles and keywords, and in the respective publications titles, per panel: A: CHE panel; B: ECO-SOC panels; C: ENG panel; D: ENV panel; E: LIF panel; F: MAT-PHY panels.

In the following figure ( Figure 4.1) we represent the correlated words frequencies observed both in projects’ titles and keywords and in the respective publications titles, per panel.

It should be noticed that in most of the scientific panels, some specific words appear more frequently, giving some scientific trends both at project and publication stages. For example, the most frequent words from the projects and linked publications in the Chemistry panel are mainly related to this scientific domain, such as synthesis, materials, drug, organic and molecular chemistry, while projects and linked publications from the Engineering panel focus more on design, model, systems, control and energy aspects. Similarly, the Environment panel shows some good correlation between the projects and linked publications mainly in environment-related keywords, such as modelling, plant, food, water, processes, energy, sustainable and industry. Interestingly, the Economics and Social Sciences panels show more interdisciplinarity with several frequent words related to health, development, care, innovation, policy and technology. Life Sciences panel shows a strong focus on cells, cancer, development, model, disease, drug, treatment and clinical aspects. Most of the frequent correlated words found in projects and linked publications from the Mathematics and Physics panels concern quantum and imaging-related keywords. Our analysis shows that in some specific scientific domains, such as quantum physics or cancer research, this tool seems to be a good predictor of what is effectively achieved and published by the projects.

**Figure 4.2.**
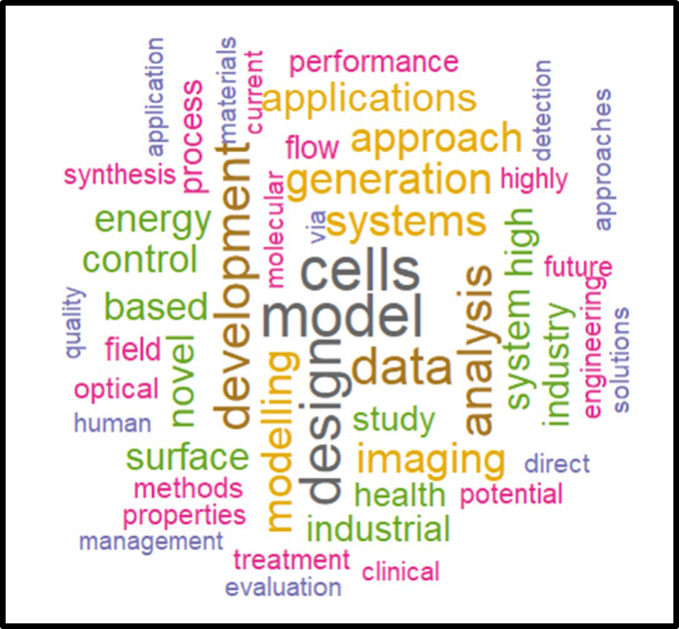
Correlated words frequencies in projects titles and keywords, and in the respective publications titles from the 118 projects flagged for innovation.

In a second phase, the same analysis was performed on the subset of the 118 projects flagged for innovation ( Figure 4.2). The results tend to show that the most innovative H2020-MSCA-ITN projects and linked publications focus on models/modelling, cells, design, data, applications, systems, energy, imaging, health, surface and industrial aspects, suggesting a significant emphasis on applied sciences, as also suggested in the HRB portfolio analysis.

In this exercise, we obtained, using a text mining analysis, a visual representation of potential thematic domains where innovative outputs can be expected, as confirmed by other analyses and indicators defined in our methodology.

### Non-tangible innovation and best practices results

Increasingly, more publications ( Rupietta *et al.*, 2018; Bader, Alharbi *et al.*, 2019) raise the importance of measuring and monitoring not only tangible innovation but also more organisational innovation elements, which can also have a crucial impact on social, environmental or policy aspects, as well as on the career development, knowledge transfer and cooperation aspects in an organisation. All these aspects are also considered as “signature” principles for MSCA. Therefore, it was important to analyse these less tangible innovation elements in the H2020-MSCA-ITN projects and investigate if some correlations could be identified with the projects reporting more tangible innovation.

The selected indicators and best practices are ranging from social innovation, innovative training practices, involvement of SME/industry in the projects to innovative supervision, networking opportunities, long-lasting collaborations, synergies and policy-driven results.

**Figure 5.1.**
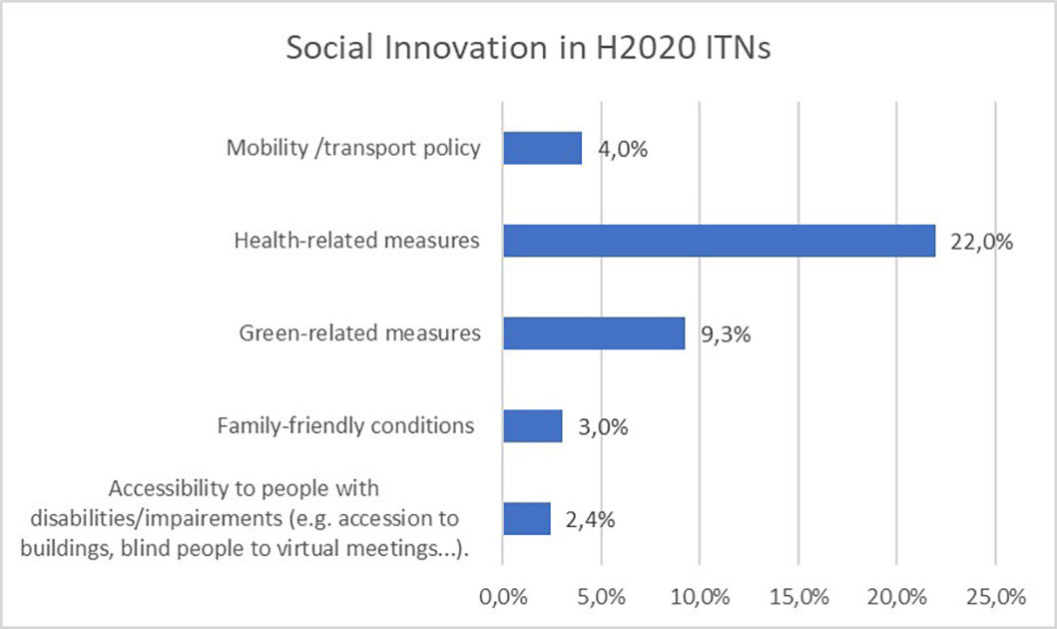
Social innovation aspects reported in H2020-MSCA 2014-2017 ITN projects.

First analyses show that for social innovation ( Figure 5.1), 22% of all 550 H2020-MSCA-ITN projects report health-related measures, followed by green-related measures (9,3%). Mobility and transport policy, family-friendly conditions or special needs/disability measures are less reported by the projects, compared to health or green-related measures. This could be explained by the recent covid-19 pandemic which impacted most of the H2020-MSCA-ITN projects, as well as by the Green Deal launched by the European Commission, encouraging the greening of all projects.

**Figure 5.2.**
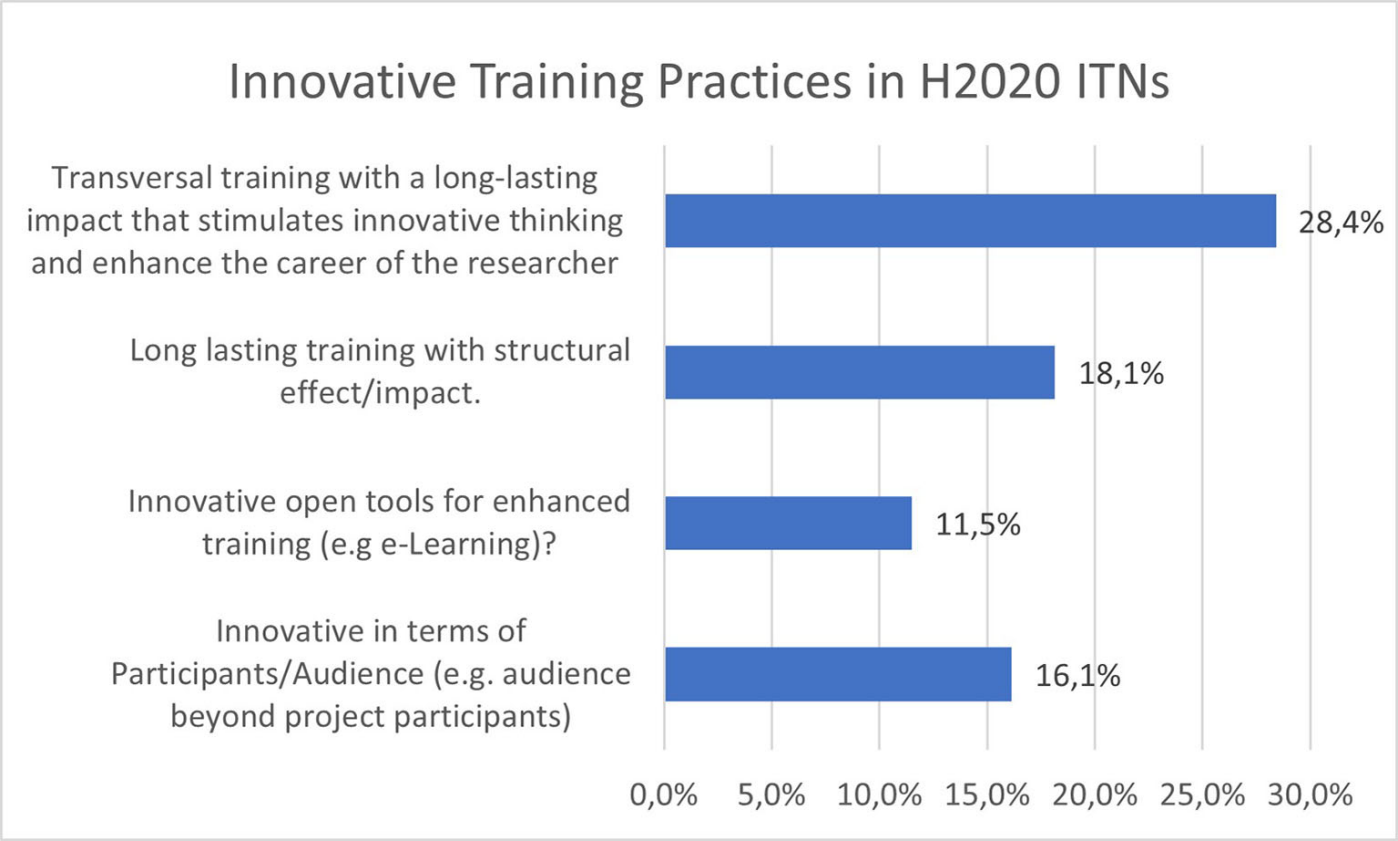
Innovative Training Practices reported in H2020-MSCA 2014-2017 ITN projects.

Innovative training practices are also a key element of H2020-MSCA-ITN projects. Interestingly, the Figure 5.2 shows that 28,4% of the projects report transversal training with a long-lasting impact that stimulates innovative thinking and enhances the researchers’ career, through for example entrepreneurial, creative thinking or teamwork aspects. An additional important element is the structural effect and impact of long-lasting training on the participating organisations, reported by 18.1% of the projects. 16.1% of the projects reported some innovation in terms of the training audience and a wider impact, while 11.5% reported innovative open tool for enhanced training, such as e-learning platforms.

**Figure 5.3.**
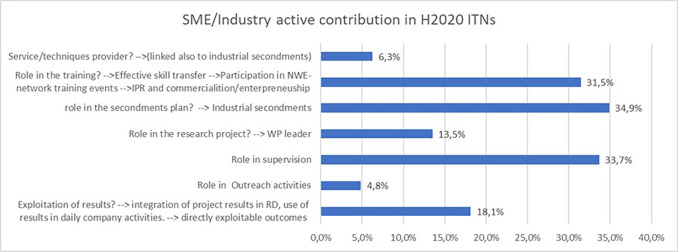
SME/industry active contribution reported in H2020-MSCA 2014-2017 ITN projects.

As reported earlier in the study, the participation of identified ‘Innovators’ plays a key role in the innovative potential of an H2020-MSCA-ITN project, a significant share of them being from the non-academic sector (in particular SME/industry). It was therefore interesting to analyse the role and active contribution of SME and industry participants in H2020-MSCA-ITN projects. The Figure 5.3 confirms that a large number of projects report a strong contribution of their SME and industry participants, in particular in the secondments of researchers, the training offered to the researchers and their supervision. Their involvement in the exploitation of results is also reported by almost 20% of the projects.

**Figure 5.4.**
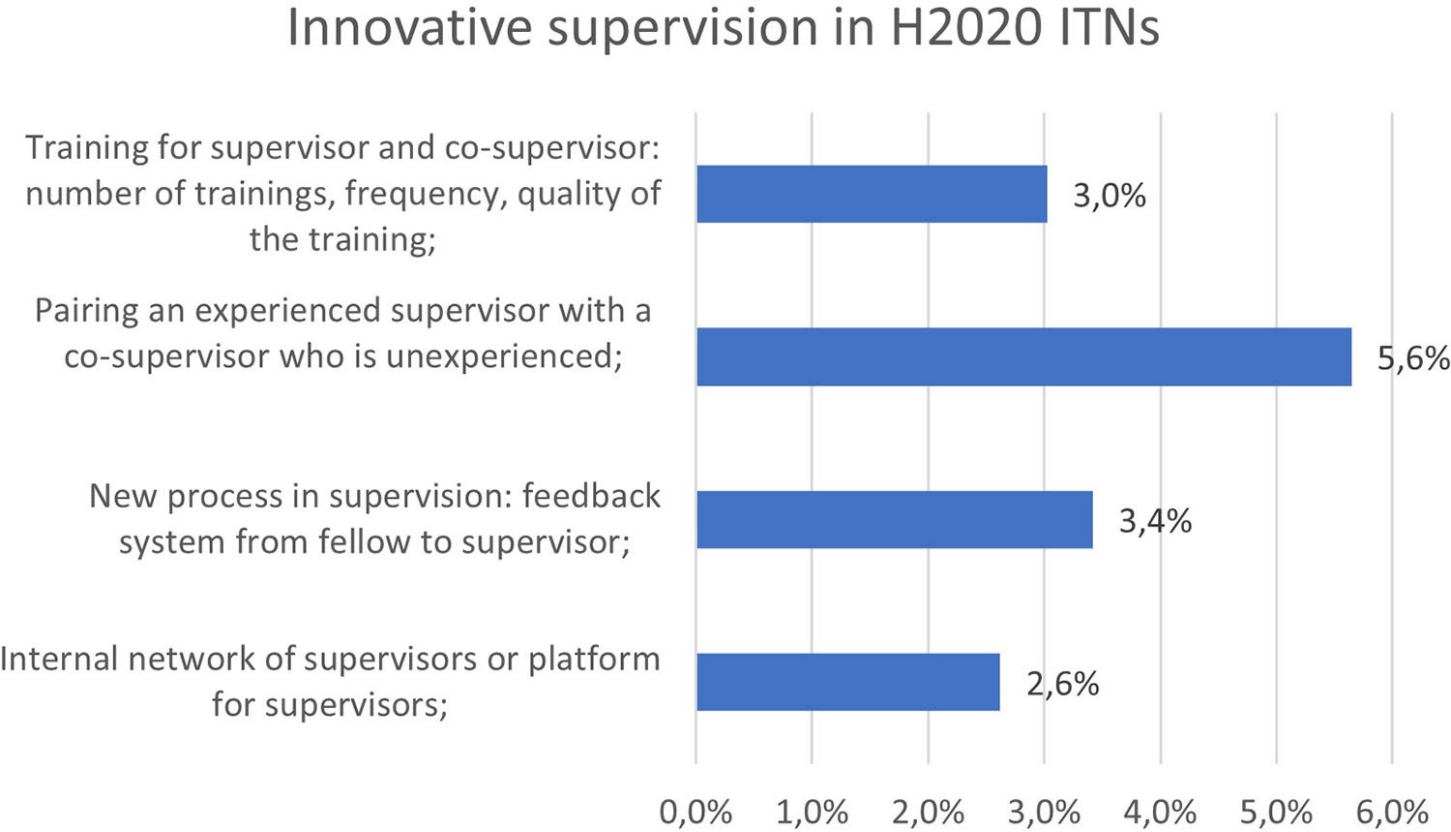
Innovative supervision aspects reported in H2020-MSCA 2014-2017 ITN projects.

Non-tangible innovation in supervision seems to be less reported by the projects ( Figure 5.4). It should be noted, however, that some projects showed a strong engagement in improving those crucial aspects in the researchers’ training and described very good practices, such as the pairing of experienced and new supervisors, dedicated training of supervisors, a new feedback system from fellow to supervisor, or an internal network or platform of supervisors to exchange knowledge and best practices. Better results could be expected at the end of H2020 and under Horizon Europe with the recently published MSCA guidelines on supervision ^16^.

**Figure 5.5.**
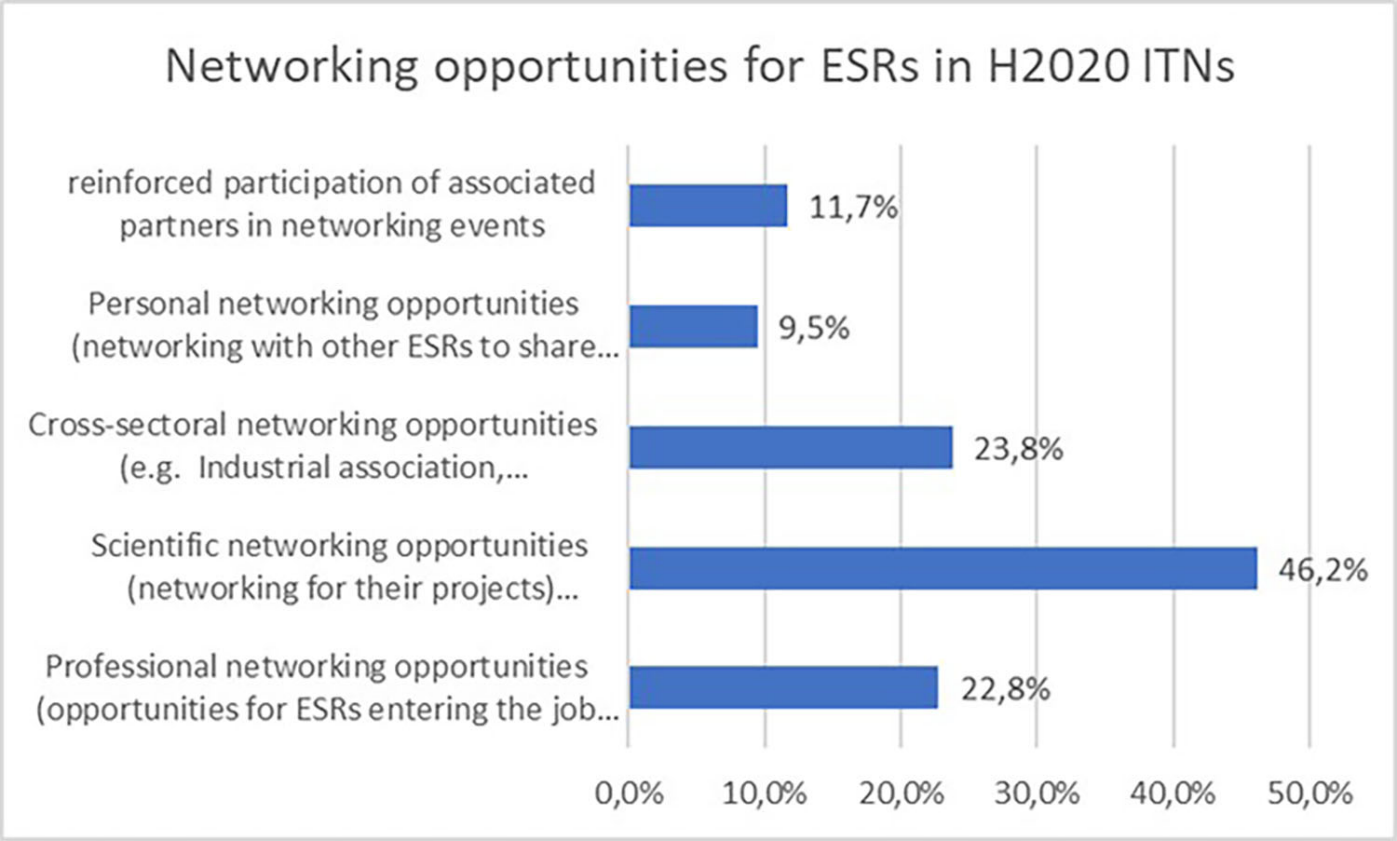
Networking opportunities reported in H2020-MSCA 2014-2017 ITN projects.

Not surprisingly, almost half of the projects report scientific networking opportunities for the ESRs, followed by cross-sectoral networking opportunities and professional networking opportunities, which is one of the objectives of the H2020-MSCA-ITN programme (see Figure 5.5).

**Figure 5.6.**
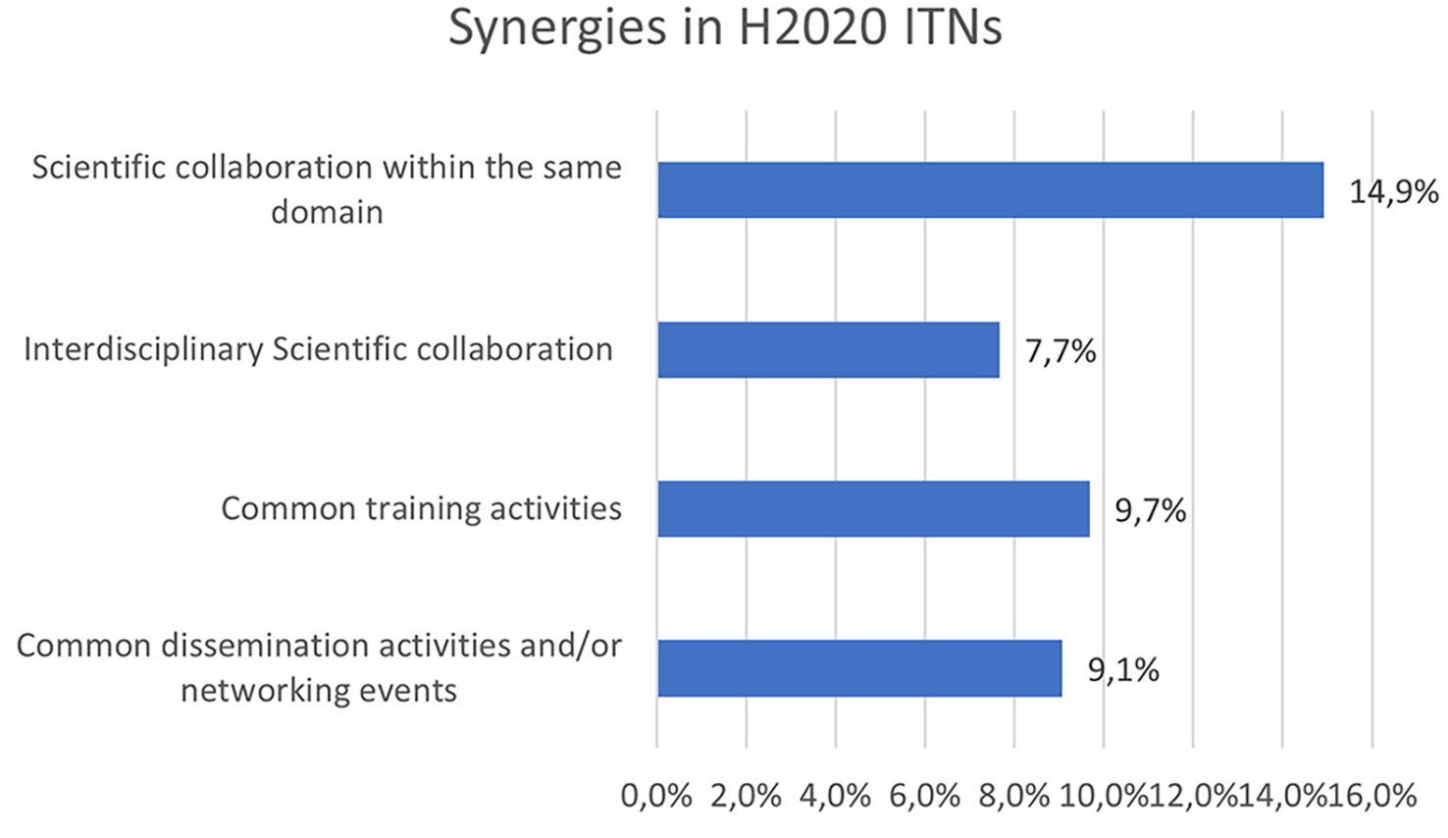
Synergy aspects reported in H2020-MSCA 2014-2017 ITN projects.

New and enhanced research results often come from synergies and synergistic effects. Synergies were also analysed in H2020-MSCA-ITN projects, as another non-tangible innovative indicator. The Figure 5.6 shows that around 15% of the projects report scientific collaboration within the same domain with other H2020-MSCA-ITN projects, while almost 10% of the projects report the organisation of common training or dissemination activities with other H2020-MSCA-ITN projects. Interdisciplinary scientific collaborations (e.g. between H2020-MSCA-ITN projects in different scientific panels) account for almost 8% of the analysed projects.

**Figure 5.7.**
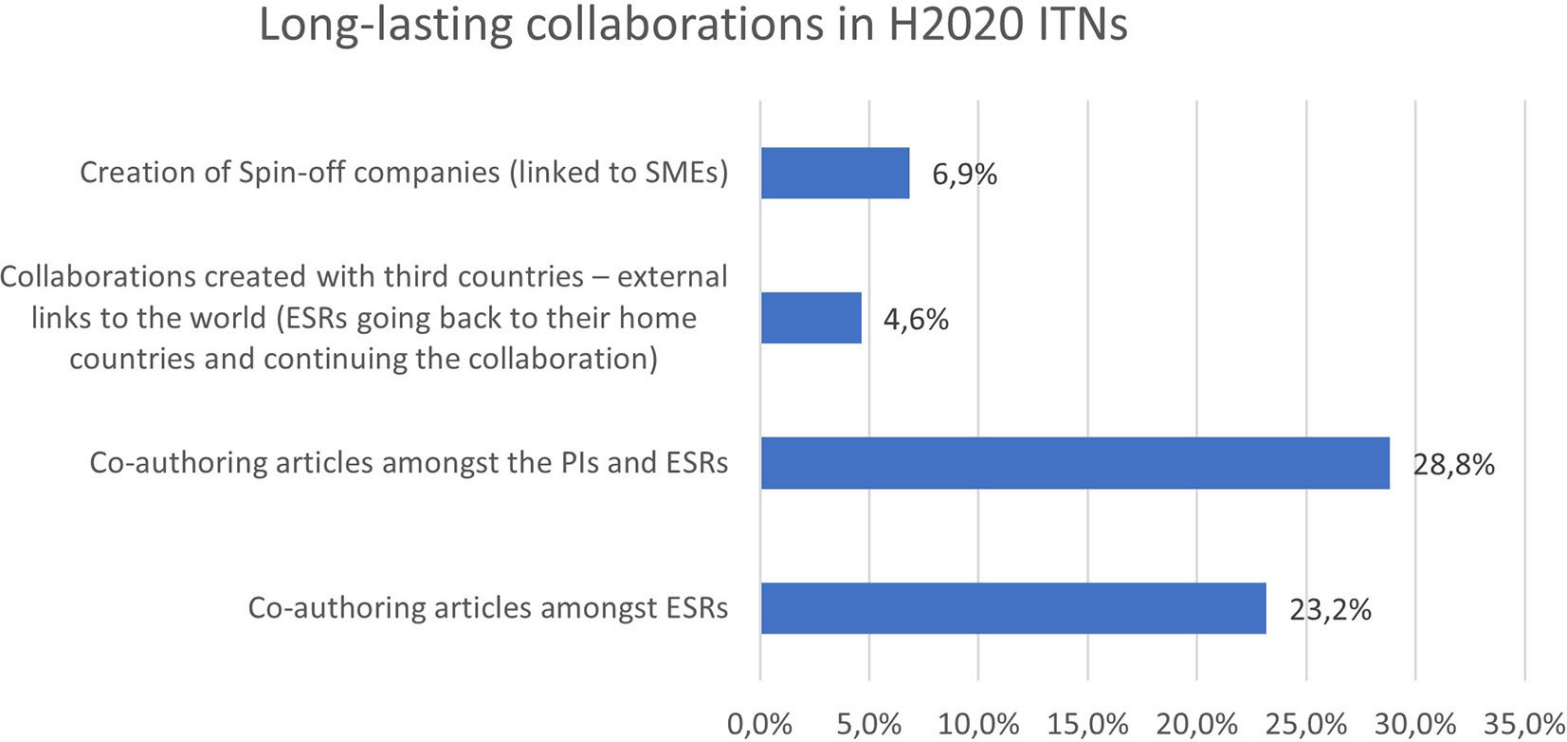
Long-lasting collaborations reported in H2020-MSCA 2014-2017 ITN projects.

Almost 30% of the H2020-MSCA-ITN projects report co-authoring articles amongst the supervisors and their ESRs. 23,2% of the projects also report co-authoring articles amongst ESRs, which is a positive result since joint publications are very much encouraged in all H2020-MSCA-ITN projects. It often demonstrates the relevance of the joint research project and how well the individual research projects, performed by the ESRs, are interconnected. In addition, the Figure 5.7 exhibits that almost 7% of the project report the creation of spin-off companies, while around 5% create strong collaborations with third countries.

**Figure 5.8.**
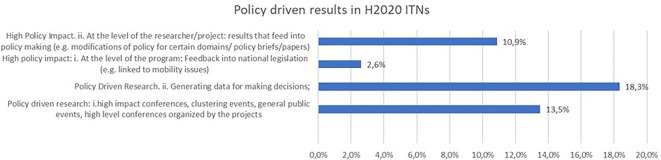
Policy driven results reported in H2020-MSCA 2014-2017 ITN projects.

Another indicator, linked to policy-driven results, was analysed in H2020-MSCA-ITN projects (see Figure 5.8). A significant share of the projects report policy-driven research, such as generating data for making policy decisions (18,3%), or organising high-impact conferences and, clustering events (13,5%). For 10,9% of the projects, the high policy impact is at the level of researcher who produces results that can feed into policy making etc …

**Figure 5.9.**
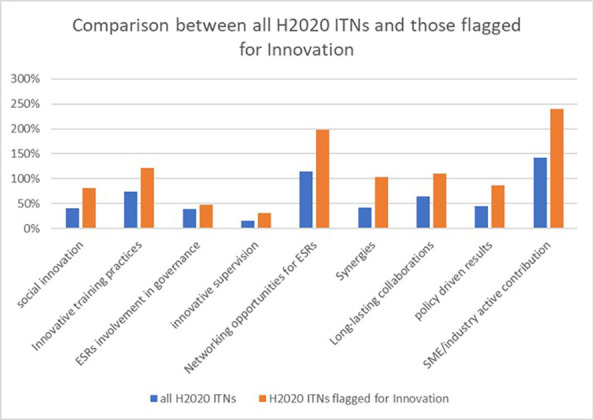
Comparison of non-tangible innovation elements and best practices between the total H2020-MSCA 2014-2017 ITN projects and the 118 projects flagged for tangible innovation. The percentages can be above 100% since projects can report more than one innovation/best practice per indicator.

Interestingly, if we compare our sample of 118 H2020-MSCA-ITN projects flagged for tangible innovation with the other H2020-MSCA-ITN projects, the Figure 5.9 below shows that the projects already reporting tangible innovation tend to also report more non-tangible innovation and best practices. The results were particularly significant for social innovation, innovative training practices or SME/Industry contribution, networking opportunities for the ESRs and synergies aspects.

### Fellows’ questionnaires

As another source of non-tangible innovation, MSCA follow-up questionnaires after the fellowship and two years after the end of the fellowship were examined.

After their fellowship, Marie Skłodowska-Curie Actions (MSCA) fellows are invited to complete two short surveys covering several aspects during and after the fellowship, including the impact of the fellowship on their skills development, career and employability. The evaluation questionnaire is completed soon after the end of their fellowship to assess their experience, skills developed and immediate next steps after their MSCA project. The follow-up questionnaire is submitted two years after the fellowship to gather further information on the more mid and long-term impact of the fellowship and the career paths of the fellows.

Regarding the questionnaires at the end of the fellowship, we also compared the respondents’ total answers (4431) with the ones from the 118 H2020-MSCA-ITN projects (1236) who declared the generation of at least one innovation or patent. The comparison of our sample of 118 H2020-MSCA-ITN projects flagged for tangible innovation with the other H2020-MSCA-ITN projects showed very similar results, as demonstrated below.

**Figure 6.1.**
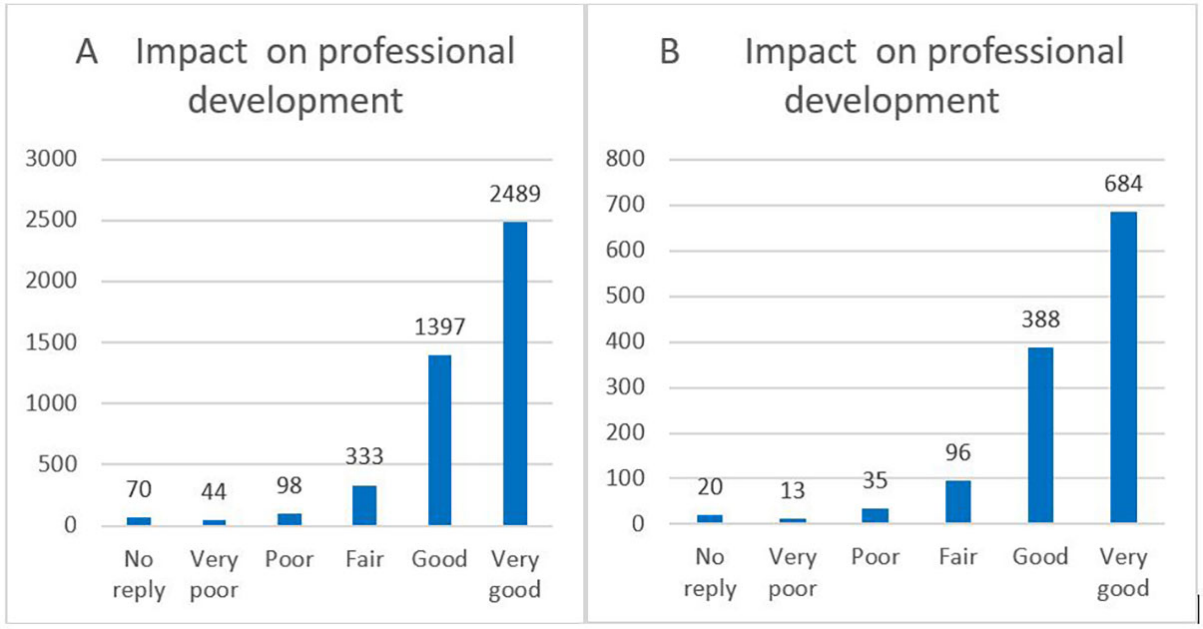
Impact on professional development. A: total replies; B: 118 projects flagged for innovation.

Recent publications highlighted several important indicators of non-tangible innovation, such as the career development and job satisfaction level of the researchers. These aspects could be analysed from the various questions included in the questionnaires. In particular, 87% of the respondents declared that their fellowship within the H2020-MSCA-ITN has had a very positive impact on their professional development (very good or good impact). The trend of replies for the 118 H2020-MSCA-ITN projects flagged for innovation was very similar to the general one (as shown in Figure 6.1 A&B).

**Figure 6.2.**
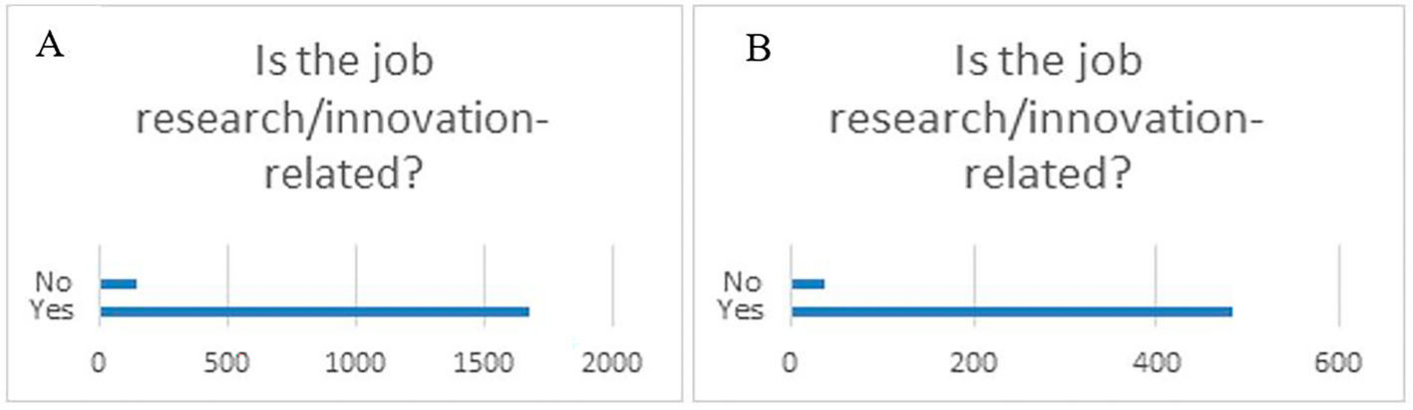
Job research/innovation-related. A: total replies (Yes: 92%; No: 8%); B: 118 projects flagged for innovation (Yes: 93%; No: 7%).

When questioned if the job was research/innovation-related, the majority of the respondents (87%), both from the overall projects sample and the 118 projects flagged for innovation, worked in a research/innovation research job after their fellowship ( Figure 6.2).

**Figure 6.3.**
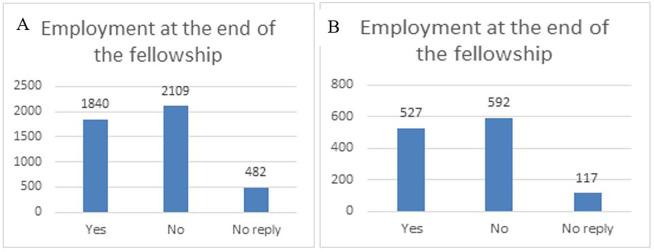
Employment at the end of the fellowship. A: total replies (Yes: 42%; No: 48%; No Reply:11%); B: 118 projects flagged for innovation (Yes: 42%; No: 48%; No Reply: 10%).

Regarding the specific impact of the H2020-MSCA-ITN fellowships on the researchers’ career, and when asking the fellows if they had found an employment at the end of the fellowship (within the first three months), a high share of fellows (around 42%) had an employment at the end of the fellowship in both samples (total H2020-MSCA-ITN portfolio and 118 projects). In general, the fellows are still finalising their PhD three months after the end of their fellowship, which can explain why almost 50% declared that they did not find an employment yet after their fellowship (see Figure 6.3). One remarkable finding is that among the 118 projects, there are 6 top performing projects, in terms of innovation and patenting where the fellows' employability is at 100% (data not shown).

**Figure 6.4.**
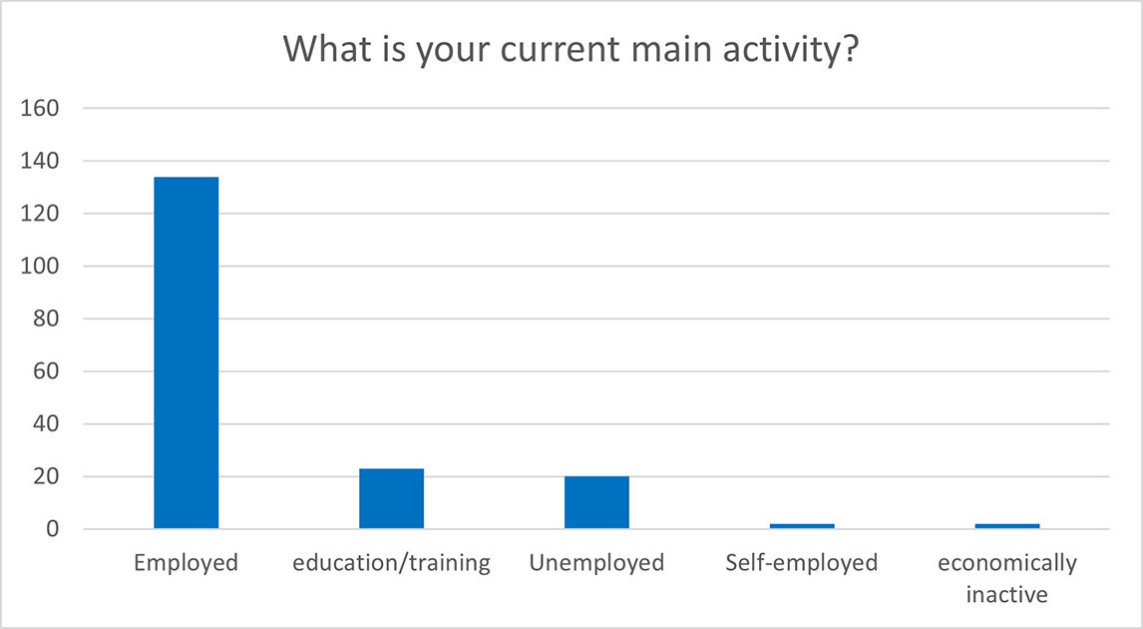
What is the current main activity of the ESRs.

The reported answers are more positive in the MSCA evaluation questionnaires two years after the end of the fellowship. In particular, out of the 181 respondents, more than 75% declared that they were employed or self-employed and 12% were still involved in education and training activities, as shown in Figure 6.4.

**Figure 6.5.**
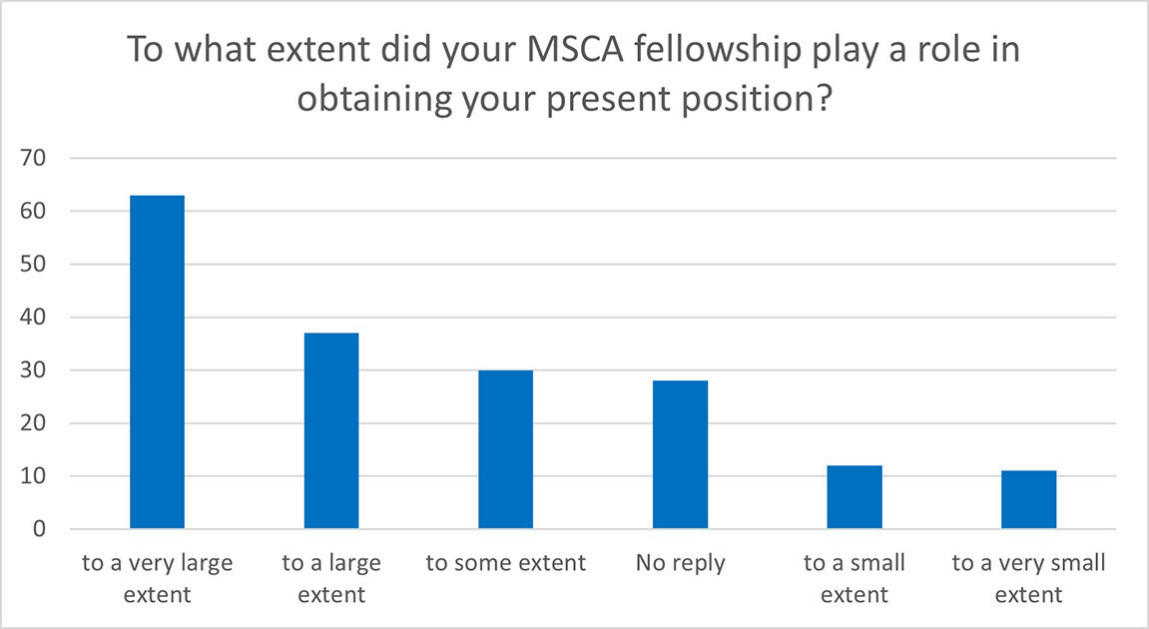
Role of the MSCA fellowship in obtaining the ESR’s current position.

The majority of the fellows considered that their MSCA fellowship played an important role in obtaining their current position. 55% of the respondents consider that their MSCA fellowship helped them to a large or very large extent ( Figure 6.5).

**Figure 6.6.**
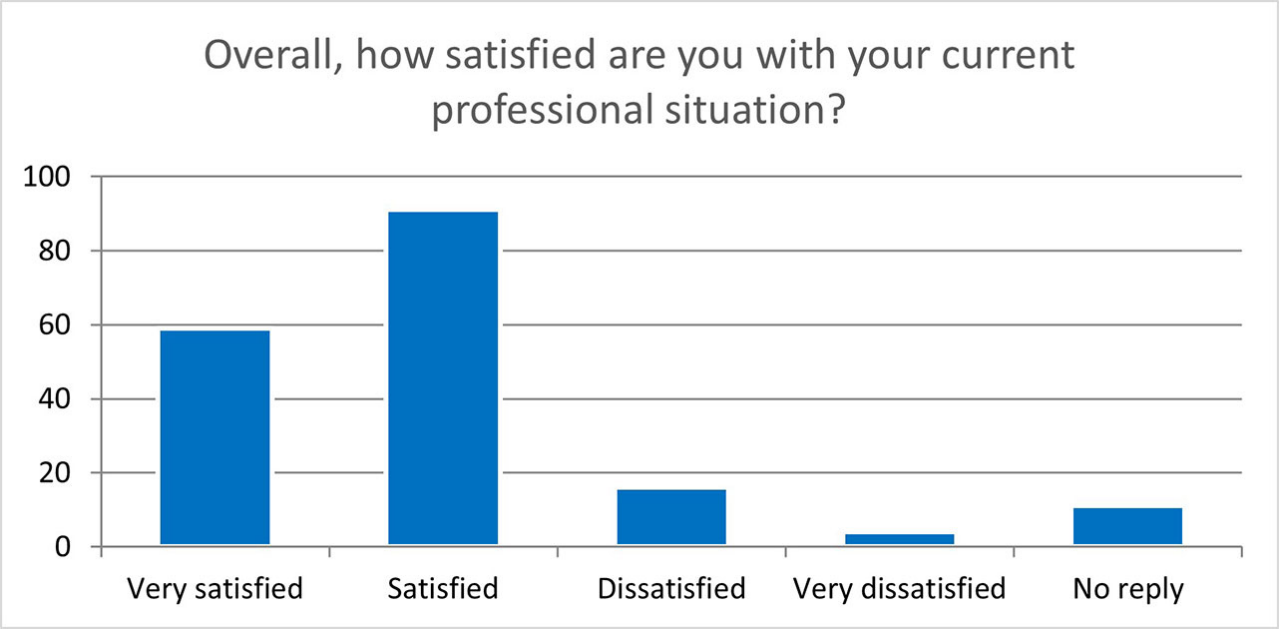
Overall satisfaction of the ESRs with their current professional situation. Very satisfied: 59 (33%); Satisfied: 91 (50%); Dissatisfied: 16 (9%); Very Dissatisfied: 4 (2%); No Reply: 11 (6%).

These results are very encouraging even for the follow-up questionnaires, two years after the end of the fellowship (181 replies), showing that most of the fellows are satisfied or very satisfied of their experience in an H2020-MSCA-ITN project and that it significantly helped them improve their professional career, as illustrated by their overall satisfaction with their current professional situation ( Figure 6.6).

**Figure 6.7.**
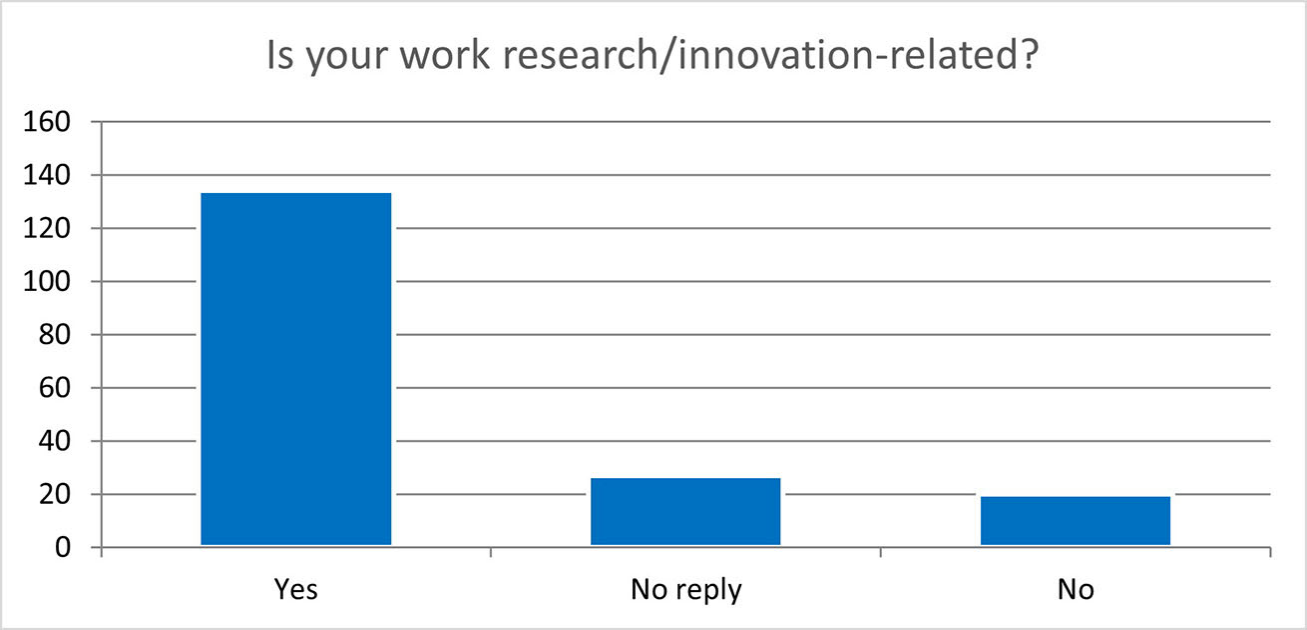
Is the ESRs’ job research/innovation-related 2 years after the end of the fellowship? Yes: 74%; No: 11%; No Reply: 15%.

When asking if their work was research/innovation-related, the same number of employed respondents (134) confirmed that their job was research/innovation-related ( Figure 6.7).

The use of questionnaires was meant to track and measure less tangible innovative outputs and notably concrete impact on the researchers’ career development through short and simple questions and appropriate indicators. It can be concluded that these types of questionnaires can be a good tool and benchmark for further improvement of MSCA work programme and policy feedback recommendations. They show how the innovative training network can enhance the employability of the fellows, especially in finding research and innovation-related jobs.

## Conclusions and recommendations

This study is, to the best of our knowledge, the first analysis proposing a methodology for tracking innovation in H2020-MSCA-ITN projects, as well as showcasing the most relevant innovative elements of H2020-MSCA-ITN projects. In order to define a methodology for tracking innovation in H2020-MSCA-ITN projects, we had to identify a representative population of H2020-MSCA-ITN projects to be analysed for innovation outputs, to identify the nature and type of data to be collected for the analysis, to propose the method to collect the necessary data, to propose and define the appropriate indicators (KPIs and metrics) in order to track, analyse and present in a quantifiable and qualitative manner innovation elements.

### Conclusions

a. In terms of the H2020-MSCA-ITN programme performance for innovation and in comparison, to the full H2020 programme, the results revealed the extent of innovative outputs and potential of the H2020-MSCA-ITN actions and confirm the excellence of the programme.
b. Even though the H2020-MSCA-ITN action is mainly a doctoral training programme for recruited researchers, the results obtained on patents and innovations revealed a high performance for the programme in the creation of such outputs. The high ratio of patents and innovation in the analysed projects, in particular in EID projects, suggests a clear added value of the allocated funding, despite the typically lower Technology Readiness Level (TRL) of the undertaken research and the early career stage of the involved researchers that are the main actors of this research in the scope of the projects.
c. From a closer analysis based on the definition of low, medium and high Innovators applied to the common participants in the H2020 and H2020-MSCA-ITN, it is noted that the percentage of SME and profit organizations is higher in the medium high Innovator group in comparison with the lower one, confirming once more, the interest of companies in developing innovation in H2020-MSCA-ITN projects as much as in H2020 programme. It is essential for the policy makers to continue to attract the non-academic sector into collaborative project consortia, to inspire/catalyse innovation outcomes and impact.
d. Patents as an indicator of potential innovation showed that certain scientific domains (e.g., pharmacology, nanoscience, physics) encompassing ‘innovation intensive industries’ are more predisposed to produce patentable results. Organisations identified as Innovators and industrial and/or commercial in their nature, are *inter alia* more likely to have better means in terms of experience and finances, and therefore may also be more likely to produce patents.
e. The clustering of the 118 projects in certain (15) scientific domains and the analysis of their innovation potential (i.e. patents, publications, innovations) indicated that certain scientific domains are more prone to generating IP-related outputs (i.e., patents). This result should be taken with caution since inventions in some domains are more patentable than others.
f. The use of word clouds, provides a visual representation of the thematic domains that are treated in the H2020-MSCA-ITN projects (based on the generated publications). This input, if complemented by an in-depth analysis, can provide interesting insights like for example, identifying domains that are less represented in terms of literature outputs and which could be further supported via future policies. Another potential field of applications can be the identification of domains that, even if they appear less-represented (in terms of publications), may however exhibit IP-related outputs (i.e. patents) or other innovative outputs, due to the nature of the examined domain (i.e. more prone to exploitation rather to dissemination). Nevertheless, this application would need further investigation to be fully validated.
g. Interestingly, the HRB analysis showed that the distribution of ‘Innovators’ that were involved in the representative sample of the 118 projects (in terms of country participation and activity type) was similar to the distribution of all participating organisations in the same sample. This can suggest that a minimum representation of proven Innovators in a given project portfolio can predict a considerable innovation output for that portfolio. Moreover, complementing this ‘mix of Innovators’ with, for example, the participation of LERU members (depending on the scientific domain) or members of similar associations can further support the generation of innovation.
h. The share of organisations considered as ‘Innovators’ among the identified 118 innovative projects, originating from countries which are defined in the European Innovation Scoreboard 2022 as ‘Emerging Innovators with performance well below the EU average’ is very low (~1.5%), while for those identified as ‘Innovation Leaders’ in the scoreboard, the share is significant (~23%). This indicates that the results of the proposed methodology in the 118 projects are aligned with the country innovation trends in the European Innovation Scoreboard 2022.
i. The HRB analysis of the 118 innovative projects showed that the majority of the projects presented a large number of events, conferences, etc. (in almost every scientific domain) which confirms the public outreach as an integral part of the H2020-MSCA-ITN programme in order to ‘transfer new knowledge to scholarly communities and communicate it to society’, in line with good practice elements for doctoral training. To a similar end, the secondments have been confirmed as an integral part of the H2020-MSCA-ITN programme (qualitative feedback provided by the participating projects in the IR questionnaires) and are considered equally important for the participating organisations and researchers, along the process of generating innovative outputs. These elements constitute an environment favourable to innovation. More specifically, the mobility of the recruited researchers and co-hosting with other researchers (for example via the secondments) tend to facilitate the co-creation of novel solutions (which are then reported as innovation outputs across several scientific domains). Similarly, the participation in wide training events or the cross-sectoral cooperation, via the exchange of researchers in academic and non-academic beneficiaries contribute to the diffusion and transfer of knowledge and the cross-sectoral cooperation, which may further stimulate innovation creation.
j. The importance of measuring and monitoring not only tangible innovation but also **more organisational innovation elements** was demonstrated in our study. We highlighted in particular the crucial impact of innovation aspects on social, environmental or policy aspects, as well as on the career development, knowledge transfer and cooperation aspects in an organisation. Interestingly, the present analysis showed that the projects already reporting tangible innovation tend to also report more non-tangible innovation and best practices. The results were particularly significant for social innovation, innovative training practices or SME/Industry contribution, networking opportunities for the ESRs and synergies. The MSCA follow-up questionnaires immediately after the fellowship and two years after the end of the fellowship also showed very positive results and a high satisfaction of the fellows, in particular on the impact of the H2020-MSCA-ITN fellowship on their career perspectives and employability. Specifically, our analysis showed that 87% of the respondents declared that their fellowship within the H2020-MSCA-ITN has had a very positive impact on their professional development. A similar trend was observed across all 118 projects and this is in line with the Marie Curie alumni association (MCAA) survey 2020 (Marie Curie alumni association survey 2020: results) where more than 80% of the respondents considered the impact of the MSCA programme in their career development as ‘very useful’ or ‘useful’.

Regarding the validation of our methodology to track, measure and analyse innovative elements in EU-funded projects, we have performed an analysis of the metrics to be considered, based on the Horizon Dashboard reports, various types of H2020-MSCA-ITN and various EC databases, focussing on **the H2020 KPIs** for high-impact publications, patents, allocated funding, innovation outputs, the **IR indicators, an analysis of most frequently found terms (words clouds),** as well as **non-tangible innovation and best practices results.** The significance of our results validates the need of having a methodology in place (as proposed in this study) to track, measure and analyse innovative aspects and best practices. Measuring innovation is key for the creation and implementation of research and innovation-friendly policies that can further boost innovation creation within the EU, and our study revealed that the H2020-MSCA-ITN funding scheme has a clear and important contribution to this process.

A set of indicators and a methodology have been proposed in this study to allow the extraction of innovative aspects (tangible and non-tangible innovation) in EU-funded projects to support policy-feedback activities. These elements can serve as a reference and can be re-used with small adaptations to other EU-funded programmes to support similar activities. The exercise for the extraction and analysis of the 118 projects confirmed the validity of the defined indicators as well as of the components of the proposed methodology, due to the coherence of results and findings which were collected via the different components of the methodology. These indicators (i.e. ‘number of Innovators participating in a given project’) can in turn act as predictors of potential success and innovation of H2020-MSCA-ITN/HorizonEurope Doctoral Networks projects or other EU-funded projects.

### Recommendations

Beyond the excellence of the results and the high performance of the programme towards innovation generation, certain points for further improvement are identified (i.e. countries representation, scientific domains currently being less-represented in the innovation generation), which can feed EU-funding policy activities to improve innovation and excellence across Europe and beyond.

a. The inclusion of ‘Innovators’ in a project consortium can act as a predictor for innovation generation. As highlighted in the HRB report, for the organisations involved in the projects, “success breeds success”. This practically means that the inclusion of significantly active organisations in a project can further build upon their capability to be even more innovative and successful, while striving to maintain a balance with newcomers. Moreover, industrial-relevant organisations, such as companies working in commercially competitive domains (i.e. pharmacological development), and which are identified as ‘Innovators’, are those being the most innovative. Therefore, by encouraging cooperation with such Innovators, project consortia can boost their success, share their expertise, their diversity and inclusiveness, and eventually their success in the generation of innovative outputs. If the ‘Innovator’ is also assigned as project coordinator, this can be an additional key success factor for realising innovation.
b. There are front-runners in innovation generation, either in terms of scientific domains or in terms of participating organisations or countries, which can be further examined, exploited or targeted by future policy-related activities, with the aim to boost innovation generation in less performing scientific domains or countries or type of organisations.
c. The share of organisations from Widening countries ^17^ among the innovative projects is significantly lower than the share of other Member States. Beneficiaries from Widening countries are thus significantly under-represented, indicating a relative lack of active engagement in H2020-MSCA-ITN projects. It should also be noted that only nine of the Innovators and none from the LERU members were from Widening countries. One explanation is that the analysis was based on the first part of H2020 projects (calls 2014-2017) and experience showed that it usually takes more time within a Framework Programme to see mid- to long- term effects of new policies, such as the H2020 goal of inclusiveness, applied to enhance participation from these countries. Involving companies and higher education institutions from these countries in the aforementioned domain of expertise, associated with the identified Innovators, should therefore be encouraged, as well as finding ways to attract and cooperate with participants from these countries in consortia with high chances of success. More targeted information days, better training by the National Contact Points, raise awareness of the partners search tool on the Funding and Tenders Portal, could be encouraged.
d. The analysis of the fellows’ questionnaires at the end of the fellowship proved to be a very interesting source of non-tangible information. It showed how the H2020-MSCA-ITN programme contributes to the employability and career development of fellows. However, the fellows’ questionnaires 2 years after the fellowship received very low attention from candidate respondents. It remains challenging to increase the response rate. Several measures could be examined to encourage the fellows’ replies to this final survey, like the use of closed-ended questions or the automatic prefilling of project-related administrative data (i.e. year, call etc.), or automatic notification to the fellow’s last known email address. These simple changes would greatly facilitate the data analysis and the accuracy of the inputs received. Coordinators could also be asked to ensure the submission of these questionnaires in due time. Additional questionnaires, targeting the coordinators could also be envisaged. This non-tangible feedback will be key to develop further policy feedback inputs.
e. In addition, the processing of project-related documents (i.e. reports, deliverables etc.) in the HRB analysis, revealed the need for further harmonisation of the information provided by the projects via the EC corporate IT tools for reporting under the current or in future funding programmes. This can be achieved by providing fixed drop-down menus for the projects to select which outputs they are reporting on. This would also help to ensure that different types of outputs receive equal amount of attention (e.g., patents and non-scientific outreach activities). The HRB report proposed specific metrics for further consideration (see “Dataset, metrics and indicators used for Horizon Results Booster analysis”).
f. In the present study, the data was collected and processed by mainly using the standard EC corporate IT tools (i.e. CORDA, IR platform and HorizonDashboard). This was done on purpose since these tools do not require specific IT competency/expertise, and our main goal was to keep the proposed methodology simple and user-friendly. When it comes to the processing of unstructured data (i.e. originated by non-tangible innovation outputs), the use of more advanced tools (even AI-related) must be considered to limit manual and time-consuming processing, involvement of external actors/experts etc.

## Data Availability

Zenodo: MSCA ITN 2014-2017 innovation and patents. https://doi.org/10.5281/zenodo.8046333 ( Bitsios *et al.*, 2023a). This project contains the following underlying data:
‐
Innovation_and_patents_2014_2017 public.pdf Zenodo: Dataset, metrics and indicators used for Horizon Results Booster analysis. https://doi.org/10.5281/zenodo.8081996 ( Bitsios *et al.*, 2023b). This project contains the following extended data:
‐ Criteria and indicators used in the HRD analysis.pdf ‐ HRB list of projects.xlsx ‐ HRB proposed metrics.pdf ‐ Project-related documents.pdf ‐ Sample data from IR.pdf Data are available under the terms of the Creative Commons Attribution 4.0 International license (CC-BY 4.0).

## Footnotes

^4^

https://ec.europa.eu/research/participants/data/ref/h2020/wp/2014_2015/main/h2020-wp1415-msca_en.pdf

https://ec.europa.eu/research/participants/data/ref/h2020/wp/2016_2017/main/h2020-wp1617-msca_en.pdf

^5^

https://research-and-innovation.ec.europa.eu/strategy/strategy-2020-2024/our-digital-future/open-science_en

^6^

https://ec.europa.eu/info/funding-tenders/opportunities/portal/screen/opportunities/horizon-dashboard

^7^

https://ec.europa.eu/eusurvey/runner/Evaluation_for_MSC_fellows

^8^

https://cordis.europa.eu/

^9^

https://ec.europa.eu/info/funding-tenders/opportunities/portal/screen/opportunities/horizon-dashboard

^10^

https://webgate.ec.europa.eu/fpfis/wikis/pages/viewpage.action?spaceKey=DIEPP&title=Horizon+Results+Booster

^11^

https://webgate.ec.europa.eu/fpfis/wikis/pages/viewpage.action?spaceKey=DIEPP&title=Horizon+Results+Booster

^12^

https://www.leru.org/

^13^

Higher and secondary education institutes (HES), Research organisations (REC), Public body (PUB), Private for profit (excluding education) (PRC), Small Medium Enterprise (SME).

^14^

Austria, Belgium, Denmark, Finland, France, Germany, Greece, Republic of Ireland, Italy, Luxembourg, the Netherlands, Portugal, Spain, and Sweden.

^15^

Bulgaria, Croatia, Cyprus, Czech Republic, Estonia, Hungary, Latvia, Lithuania, Malta, Poland, Romania, Slovakia, and Slovenia.

^16^

https://op.europa.eu/en/publication-detail/-/publication/bb02d56e-9b3c-11eb-b85c-01aa75ed71a1

^17^

Bulgaria, Croatia, Cyprus, Czech Republic, Estonia, Hungary, Latvia, Lithuania, Malta, Poland, Romania, Slovakia, and Slovenia.
