# Supplementary material for: The innovative dimension of the research training programmes under H2020-MSCA-ITN: a methodological approach to track, measure and analyse innovative aspects and provide policy-feedback conclusions
Source: F1000Res. 2023 Oct 26;12:1020. Originally published 2023 Aug 22. [Version 2] doi: 10.12688/f1000research.138482.2 (PMC10523102; doi:10.12688/f1000research.138482.2)
Supplement: Peer review history

# Reviewer report: Reviewer response for version 2

**DOI:** 10.5256/f1000research.156824.r218443

**Published:** 2023-11-03

**Authors:** Nikolaos D. Alexopoulos

**Affiliations:** 1 Department of Financial Engineering, School of Engineering, University of the Aegean, Chios, Greece

The authors responded well to the suggested comments by the reviewers. They also added Figure 0.1 to visually reflect the steps of the proposed methodology, as sugegsted by the reviewers. The article is well ammended with the revised version and I recommend to accept the manuscript with its present form.

Is the work clearly and accurately presented and does it cite the current literature?

Yes

If applicable, is the statistical analysis and its interpretation appropriate?

Yes

Are all the source data underlying the results available to ensure full reproducibility?

Yes

Is the study design appropriate and is the work technically sound?

Yes

Are the conclusions drawn adequately supported by the results?

Yes

Are sufficient details of methods and analysis provided to allow replication by others?

Yes

Reviewer Expertise:

innovation; materials science; engineering; economics and business

I confirm that I have read this submission and believe that I have an appropriate level of expertise to confirm that it is of an acceptable scientific standard.

# Reviewer report: Reviewer response for version 2

**DOI:** 10.5256/f1000research.156824.r218444

**Published:** 2023-10-30

**Authors:** Costis Kompis

**Affiliations:** 1 Vodera Ltd., London, UK

The implemented improvements in both the text and the new image are approved.

Is the work clearly and accurately presented and does it cite the current literature?

Yes

If applicable, is the statistical analysis and its interpretation appropriate?

Yes

Are all the source data underlying the results available to ensure full reproducibility?

Yes

Is the study design appropriate and is the work technically sound?

Yes

Are the conclusions drawn adequately supported by the results?

Yes

Are sufficient details of methods and analysis provided to allow replication by others?

Yes

Reviewer Expertise:

Technology commercialisation from R&D, integrating science & engineering with innovation management. Technology domains span Cyber Physical Systems, Data Analytics, Machine Learning, Energy Harvesting, Virtual Reality, Medical Imaging, Fuel Cells and Additive Manufacturing.

I confirm that I have read this submission and believe that I have an appropriate level of expertise to confirm that it is of an acceptable scientific standard.

# Reviewer report: Reviewer response for version 2

**DOI:** 10.5256/f1000research.156824.r218442

**Published:** 2023-10-30

**Authors:** Alessandro Marino

**Affiliations:** 1 Department of Electrical and Information Engineering “Maurizio Scarano”, University of Cassino and Southern Lazio, Cassino, Lazio, Italy

Thanks to the authors for having answered all my comments. I have no further comments.

Is the work clearly and accurately presented and does it cite the current literature?

Yes

If applicable, is the statistical analysis and its interpretation appropriate?

Yes

Are all the source data underlying the results available to ensure full reproducibility?

Partly

Is the study design appropriate and is the work technically sound?

Yes

Are the conclusions drawn adequately supported by the results?

Yes

Are sufficient details of methods and analysis provided to allow replication by others?

Yes

Reviewer Expertise:

Automation and Robotics. Computer Science Engineering.

I confirm that I have read this submission and believe that I have an appropriate level of expertise to confirm that it is of an acceptable scientific standard.

# Reviewer report: Reviewer response for version 1

**DOI:** 10.5256/f1000research.151683.r202225

**Published:** 2023-09-28

**Authors:** Alessandro Marino

**Affiliations:** 1 Department of Electrical and Information Engineering “Maurizio Scarano”, University of Cassino and Southern Lazio, Cassino, Lazio, Italy

First and foremost, I would like to express my very positive opinion about the initiative. As reported by the authors, monitoring the results of the project, particularly within a program as extensive as the H2020-MSCA-ITN program, is noteworthy and of the utmost importance. This step is fundamental for highlighting weaknesses and strengths of the aforementioned program and for taking corrective actions if needed. It is also remarkable that both tangible and non-tangible outputs are considered, as the former often fail to capture many relevant features of the phenomena under study, as highlighted in the literature.

 Furthermore, another fundamental contribution of the proposed approach is that the underlying methodology might also apply to other programs.

 Finally, it is very positive that the MSCA program has performed very well and has fulfilled almost all of its objectives.

 Having said that, here are a few comments:

1. After the introduction of background and method, instead of using items and standard text format, a table summarizing the main features and indicators considered and the corresponding database would further enhance the document's readability.
2. The identified scientific domains make complete sense, though more details might be provided on how they were identified.
3. The comparison between MSCA and the remaining H2020 programs is remarkable. The reader is wondering if RIA and IA could be considered separately in future comparisons given the different research components present in the two typologies, the different TRL targets, and often the different presence of industries in the Consortia. Does it make sense to compare MSCA-EID to H2020-IA and other MSCA programs with H2020-RIA?
4. Regarding produced publications, they might be classified in future studies based on the ranking of the corresponding journal/conference. For example, you could use the SJR ranking for journals and conference ranking or acceptance rate for conference papers, as well as citations.
5. The article appropriately highlights the limitations of the analysis due to the available data and their format and makes useful recommendations at the end of the document. I believe that a potential follow-up to the present study might involve a deeper investigation into what data (especially for non-tangible outputs) are worth gathering in addition to the ones already collected and how they can be gathered to ease the processing phase.
6. Regarding footnotes: Links 9-11 are not accessible in my case.

In summary, I would strongly support the indexing of the given document since it presents elements of novelty in the evaluation of complex programs and serves as an example of good practice.

Is the work clearly and accurately presented and does it cite the current literature?

Yes

If applicable, is the statistical analysis and its interpretation appropriate?

Yes

Are all the source data underlying the results available to ensure full reproducibility?

Partly

Is the study design appropriate and is the work technically sound?

Yes

Are the conclusions drawn adequately supported by the results?

Yes

Are sufficient details of methods and analysis provided to allow replication by others?

Yes

Reviewer Expertise:

Automation and Robotics. Computer Science Engineering.

I confirm that I have read this submission and believe that I have an appropriate level of expertise to confirm that it is of an acceptable scientific standard.

# Response

**Published:** 2023-10-05

**Authors:** IOANNIS BITSIOS

**Affiliations:** MSCA-Doctoral Networks, EUROPEAN RESEARCH EXECUTIVE AGENCY (REA), Brussels, Belgium

Dear Reviewer, thank you very much for your useful feedback and pertinent comments. We addressed most of your suggestions. An updated version of the article will be soon made available.

 Best regards,

 Ioannis, Fabrizio, Riccardo and Audrey

# Reviewer report: Reviewer response for version 1

**DOI:** 10.5256/f1000research.151683.r202218

**Published:** 2023-09-26

**Authors:** Nikolaos D. Alexopoulos

**Affiliations:** 1 Department of Financial Engineering, School of Engineering, University of the Aegean, Chios, Greece

In this study, an innovative methodological approach is proposed for tracking and analyzing innovation and representative indicators were considered from the applicable Multi-Annual Framework Programme Horizon 2020. The article provides first-level input for policy-feedback activities, identifies the scientific domains and the EU countries that urge for innovation generation. The writing style is appropriate, Figures and Tables support the conclusions and are appropriate.

 The manuscript is worth indexing and I recommend it being accepted after some minor amendments:

1. The Abstract should be essentially shortened. Especially for background and methods, only the basic information should be reported in this section. The focal point should be the results/conclusions of the present study.
2. Citing style should be re-considered; there are some entries as footnotes (starting from no4); they should be considered as regular citations and follow the style approved by the journal.
3. It is recommended (not mandatory) that the authors to provide a Figure just like a process flow diagram that briefly shows the logical path and the sequence of the intermediate steps of their study.
4. Figures: The figures should be numbered in sequence, 1, 2, 3 as it is extremely inconvenient for the reader to follow Figure e.g., 1.b.1 or 5.1 or 3.b.10.
5. The captions in the figures should be more descriptive, i.e., it should describe the Figure to the reader.
6. The conclusions should be essentially shortened; there are ten (10) numbered conclusions in the manuscript; each numbered conclusion is almost a paragraph long and this is not friendly for the readers. The conclusions should be shortened to simply report the key results of the manuscript. The discussion of the key factors/results should be transferred to the discussion section of the manuscript.

Is the work clearly and accurately presented and does it cite the current literature?

Yes

If applicable, is the statistical analysis and its interpretation appropriate?

Yes

Are all the source data underlying the results available to ensure full reproducibility?

Yes

Is the study design appropriate and is the work technically sound?

Yes

Are the conclusions drawn adequately supported by the results?

Yes

Are sufficient details of methods and analysis provided to allow replication by others?

Yes

Reviewer Expertise:

innovation; materials science; engineering; economics and business

I confirm that I have read this submission and believe that I have an appropriate level of expertise to confirm that it is of an acceptable scientific standard.

# Reviewer report: Reviewer response for version 1

**DOI:** 10.5256/f1000research.151683.r202223

**Published:** 2023-09-20

**Authors:** Costis Kompis

**Affiliations:** 1 Vodera Ltd., London, UK

This a well-structured, erudite and well-informed article. Its methodology and analysis benefit from rich data sets held in EC databases. The methodology is practical and appropriately leverages the available data. There are references to recent relevant works (including HRB report), which set strong foundations. Granular descriptions of the indicators are given. The conclusions are meaningful and can certainly have implications in the design and execution of future programmes for training new generations of researchers and entrepreneurs. 

 Below, I append the issues - organised per the article's sections - that I identified along with possible reformulations and other suggestions that the authors can take into consideration in order to improve clarity and consistency. 

**Abstract:**

- “for the forging of highly skilled” => for forging highly skilled.
- examined and considered => considered and examined.
- questionnaires/surveys findings => questionnaires/surveys’ findings.

**Introduction:**

- "The low success rate": “low” is relative. For other competitions 7-8% might be considered “high”. I suggest to simply replace “the low” with “a”.  
- “All these networks constitute a potential gold mine” => “The set of these networks potentially represents a gold mine”.  
- "The action’s performance": please improve clarity to what “action” exactly refers to here. Perhaps just say H2020-MSCA-ITN or should it be introduced once and referred with a capital A throughout the article?   

**Background:**

- "H2020-MSCA-ITN being the EU’s flagship scheme for establishing transnational doctoral programmes, it is of interest [...]" => "H2020-MSCA-ITN, being the EU’s flagship scheme for establishing transnational doctoral programmes, is of interest [...]."
- “As also reflected in the concept note that kickstarted the present study, the MSCA-ITN projects can constitute a potential resourceful place for innovation.”: This statement does not seem necessary and could be dropped.   
- “‘technology’ innovation” => technological innovation (there are additional references of this term in the rest of the article that may need to be edited should the change be agreeable).
- “provide a methodology” => ”propose the methodology”.

**Methods:**

- “proposed and defined the appropriate indicators (KPIs and metrics)”: how is “appropriate” qualified here? Perhaps replace it with ‘suitable’ meaning that they are fit for the purpose of measuring innovation in such type of EU-funded projects.
- “appear to have generated some kind of innovation or patent”: Consider if the wording can be improved, e.g. “self-declare the generation of innovations, including the filing of patents.”
- “the best and promising candidate”: replace simply with something like that “the sample was **suitable**”?
- “applied to the MSCA” => “applied it to the MSCA”.
- “Two indicators based on the Innovation Radar data (Innovation Radar, 2018) have been defined”: it is not clear if it is the authors of the Innovation Radar or yourselves who defined the indicators from this statement. If it is you, please make it more explicit, i.e. “Two indicators defined from the Innovation Radar data (Innovation Radar, 2018) have been selected as more suitable for our analysis:”
- “In formal terms,” => “Specifically,” or ‘Typically,’?
- “We have extracted” => "We extracted".
- “These experts were provided [...]”: this sentence appears fragmented with a bit of repetition “which had been already extracted” and “which have been extracted”. Please consider linking these explanations to make it more concise. For example, “These experts were provided with the necessary data from the 118 projects (innovations and patents), the IR reports/questionnaires (extracted by the IR platform) and with additional project-related documents, like reports, deliverables, description of work, etc. (extracted from the EC corporate databases), resulting in thousands of documents to be considered and analysed for the total of 118 projects.”
- “follow-up questionnaires after the fellowship” => “follow-up questionnaires immediately after the fellowship” (in order to make a distinction with the “and two years after the end of the fellowship” that follows?).  
- "to errors (typos, clerical errors)" => "to typos or clerical errors".
- "A methodology and pre-defined categories were discussed in order to limit as much as possible the subjectivity and the variability in the data collection exercise." => "Our methodology and introduction of the pre-defined categories aimed at reducing the subjectivity and the variability in the data analysis phase."
- This can be considered as expected for two reasons: The generation of patents: can use i) and ii) to make it more clear.
- "may vary." => "exhibited variability."    
- "as more relevant." => "more relevant."
- “It must be noted” => "Notably"
- “This can be an encouraging early finding on the effect of the project mode (EID, more industry-oriented) to the generation of patents.”: Not sure about what “encouraging” implies here. Better stay factual saying something like “This is consistent with the expectation that EIDs which are more industry-oriented prioritise the generation of patents."
- “same innovations character”: reconsider the wording here. Perhaps “similar capacity to generate innovation”? 
- “However, it could be expected if considering the following analysis of the common innovators in both populations as follow-up of the HRB study.”: This sentence could be dropped, not least because it creates an anti-climax after “truly remarkable achievement”.    
- "the higher the innovators ICI" => "the higher the innovators’ ICI" (apostrophe was missing).
- “This is clearly visible in the pie charts for each innovators category.” => "as shown in the relevant Figures". 
- "This is somehow expected" => "This is not surprising".
- "innovation character" => "innovator character/type" (to comply with the terminology used by the Innovation Radar).
- "ICI and the higher is the percentage of non-profit organisation" => "ICI, the higher is the percentage of non-profit organisations".
- “some are more prolific in this regard than others." => “Some are more prolific in this regard than others.” 
- “Finally, and": could be omitted.   
- "(as part of the analysed documents)": could be omitted.
- "the participation of identified innovators plays" => "the participation of identified 'Innovators' plays".
- “In the context of more and more requests of feedback to policy, “: Is this necessary? If yes, it’d be better to explain who makes the requests etc.
- "For 10,9% of the project" => "For 10,9% of the projects".
- “ etc …”: omit?  
- "When asking" => "When questioned".
- Figure 6.3. Employment at the end of the fellowship. A: total replies (Yes: 42%; No: 48%; No Reply:11%): Check the rounding as the answers add to 101%.    
- "therefore it" => "which".
- “6 very best projects”: please use an alternative descriptor. Perhaps “top performing in terms of innovation and patenting”.
- "Even more positive results could be observed when analysing the MSCA evaluation questionnaires two years after the end of the fellowship." => "The reported answers are more positive in the MSCA evaluation questionnaires two years after the end of the fellowship."  
- Figure 6.7. Is the ESRs’ job research/innovation-related 2 years after the end of the fellowship? Yes: 74%; No: 11%; No Reply: 15%. => Whether the ESR’s job is research/innovation-related 2 years after the end of the fellowship. (Yes: 74%; No: 11%; No Reply: 15%).  
- "we had to identify the appropriate population" => "we had to identify a representative population". 
- “in the MSCA-ITN projects.” (end of par on. p29): can be omitted.  
- "the results reveal the" => "the results revealed the" (or better “the results revealed the extent”).
- “while additional interesting insights emerged”: can be omitted.  
- "Revealed a high performance of the programme" => "Revealed a high performance for the programme".
- “the perceived low TRL”: “typically lower TRL”? (you may have to expand the acronym or add a footnote for any reader not familiar with TRLs).
- “to ensure successful innovation outcomes”: not sure about “successful” in this sentence. Perhaps “to inspire/catalyse innovation outcomes”.  

**Conclusions:**

- “to generate a high number of patents.”: or perhaps rephrase it that inventions in some domains are more patentable than others.
- “This can suggest that a sufficient mix of innovators in a given project portfolio can predict a considerable innovation output for that portfolio.”: possibly but hard to prove causality as such. In any case I would suggest reformulation to avoid vague descriptors such as “sufficient mix”. Perhaps instead you want to mention a “minimum representation of proven “Innovators””.  
- “European Innovation Scoreboard 202”: something missing at the end.
- “nurture” => “tend to facilitate”? 
- “which further stimulate innovation creation” => "which may further stimulate innovation."
- "The importance to measure and monitor" => "The importance of measuring and monitoring".
- break the first sentence (e.g. put a full stop after “in our study”) to improve readability.
- "synergies aspects" => "synergies".
- "after the fellowship and two years after the end of the fellowship" => "immediately after the fellowships and two years after the end of the fellowships".
- "Indeed" => "Specifically".
- "A similar trend was found for the 118 projects. Moreover, a similar result was also reported in the Marie Curie alumni" => "A similar trend was observed across all 118 projects and this is in line the Marie Curie alumni".
- “since coherent results and findings were observed across the components of the methodology.” => "due to the coherence of results and findings collected via the different components".
- “These indicators can in turn act as predictors of potential success and innovation of ITN/DN projects or other EU-funded projects.“: Unclear why the term “predictors” is used here in reference to “a set of indicators and a methodology”. Perhaps you can list separate indicators that lend themselves to such predicting capability. One of them can be the number of innovators participating in a given project.
- “a right balance”: not obvious to what that might be. If there is a proven mix, please indicate it even as a percentage. If not, simply replace with “balance”.  
- “innovation front-runners” => “front-runners in innovation generation”?
- "could also be envisaged." => "could be encouraged."?

**General/consistency issues:**

- For consistency, please use EU-funded (there are several instances of EC-funded too).
- Innovation Radar/IR: term introduced but acronym not used consistently thereafter.
- “MSCA ITN projects from the call years 2014 to 2017” vs “2014-2017 ITN Projects” (e.g. p 9).
- “Marie Skłodowska-Curie Actions (MSCA)” appears on p. 26. Shouldn’t be in first occurrence? 

Is the work clearly and accurately presented and does it cite the current literature?

Yes

If applicable, is the statistical analysis and its interpretation appropriate?

Yes

Are all the source data underlying the results available to ensure full reproducibility?

Yes

Is the study design appropriate and is the work technically sound?

Yes

Are the conclusions drawn adequately supported by the results?

Yes

Are sufficient details of methods and analysis provided to allow replication by others?

Yes

Reviewer Expertise:

Technology commercialisation from R&D, integrating science & engineering with innovation management. Technology domains span Cyber Physical Systems, Data Analytics, Machine Learning, Energy Harvesting, Virtual Reality, Medical Imaging, Fuel Cells and Additive Manufacturing.

I confirm that I have read this submission and believe that I have an appropriate level of expertise to confirm that it is of an acceptable scientific standard.
